# Evidence on artificial intelligence-assisted clinical documentation and healthcare workers’ emotional wellbeing at work: a scoping review

**DOI:** 10.3389/fpsyg.2026.1840884

**Published:** 2026-06-24

**Authors:** Na Xiao, Li He, Lan Chen, Sengtong Liu, Qian Xiang, Pingping Wang

**Affiliations:** 1 Healthcare-Associated Infection Control Center, Sichuan Academy of Medical Sciences, Sichuan Provincial People's Hospital, School of Medicine, University of Electronic Science and Technology of China, Chengdu, Sichuan, China; 2 Hospital Infection Control Department, Sichuan Provincial People's Hospital-Pujiang People's Hospital, Chengdu, Sichuan, China; 3 Irradiation Preservation Key Laboratory of Sichuan Province, Department of Planning, Finance, and Quality Assurance, Chengdu Institute of Food Inspection, Chengdu, Sichuan, China; 4 School of Medicine, University of Electronic Science and Technology of China, Chengdu, Sichuan, China

**Keywords:** artificial intelligence, clinical documentation, emotional wellbeing, healthcare workers, scoping review

## Abstract

**Objective:**

This scoping review mapped evidence on artificial intelligence-assisted clinical documentation for healthcare workers’ emotional wellbeing at work, including tool types, reported favorable, adverse, or mixed findings, and evidence gaps. We also considered how these tools may shape documentation-related work demands, autonomy, clinical voice, patient connection, and broader occupational wellbeing.

**Methods:**

We searched PubMed, Web of Science, Embase, CINAHL, and PsycINFO from database inception to March 16, 2026, to identify studies. The review was conducted in accordance with the Joanna Briggs Institute methodological framework and reported following the Preferred Reporting Items for Systematic Reviews and Meta-Analyses Extension for Scoping Reviews guidelines. Two reviewers independently conducted study selection and data extraction. The findings were synthesized using descriptive and narrative approaches, and the included studies were critically appraised using the Mixed Methods Appraisal Tool 2018.

**Results:**

Thirty-five studies met the inclusion criteria. The evidence base was highly concentrated: 31 of the 35 studies were conducted in the United States, and 30 evaluated ambient artificial intelligence scribe tools. Overall, 23 studies reported predominantly favorable findings, while 12 were classified as reporting mixed findings. The most frequently reported benefits included reduced documentation burden, decreased cognitive load, improved work satisfaction, and better perceived patient connection. Outcomes related to burnout were highly variable. Mixed findings were primarily associated with implementation barriers, the need for editing, accuracy concerns, and challenges related to preserving clinical voice and professional autonomy.

**Conclusion:**

Current evidence indicates that artificial intelligence-assisted clinical documentation is mainly associated with clinician-reported relief in documentation-proximal strain, especially perceived documentation burden and cognitive load. However, the evidence remains concentrated in early evaluations of ambient artificial intelligence scribes in United States healthcare settings and should not be generalized to all documentation artificial intelligence tools or health systems. Findings for broader emotional wellbeing outcomes, including burnout, remain limited and mixed. Given the methodological concerns identified in the Mixed Methods Appraisal Tool appraisal, these findings should be interpreted as reported associations and perceived changes rather than causal evidence. Future studies should use longitudinal, multicenter designs and validated wellbeing measures to assess durability, safety, and longer-term occupational outcomes.

**Systematic review registration:**

https://osf.io/m8e9v.

## 1 Introduction

Clinical documentation is a fundamental component of healthcare delivery, supporting clinical communication, continuity of care, patient safety, legal accountability, and reimbursement (Ebbers et al., 2022; Seligson et al., 2021). Despite its essential role, however, documentation has long been recognized as a major source of burden for healthcare workers (Moy et al., 2021; Gesner et al., 2022). Healthcare workers routinely spend substantial time drafting, editing, and structuring clinical notes, often under considerable time pressure and frequently beyond scheduled working hours (Pearlman et al., 2025; Rotenstein et al., 2024). With the expansion of electronic health records, regulatory requirements, and performance-oriented healthcare systems, documentation has come to occupy an increasingly large share of everyday clinical work and has become a persistent source of workplace strain (Doherty et al., 2025; Murad et al., 2024; Levy et al., 2024).

Documentation burden has been linked to a range of adverse occupational outcomes, including burnout, stress, emotional exhaustion, job dissatisfaction, and work-life imbalance (Wu et al., 2024; Attipoe et al., 2023; Holmgren et al., 2024; Frintner et al., 2021). Its consequences are not limited to the time required to complete notes. The concurrent demands of documentation and patient care may increase cognitive load, fragment attention, and reduce healthcare workers’ sense of presence during clinical encounters (Asgari et al., 2024; Melnick et al., 2020). The need to move continuously between listening, reasoning, documenting, and meeting administrative expectations may also diminish perceived autonomy and weaken the relational and meaningful aspects of care (Melnick et al., 2020; Moy et al., 2023). Documentation should therefore be understood not only as an administrative responsibility, but also as a workplace condition that can shape healthcare workers’ psychological experience of work. In this sense, artificial intelligence (AI)-assisted clinical documentation is relevant not only as a technical or efficiency intervention, but also as a change in how clinical work is organized and experienced. By shifting part of note production to AI-supported systems, these tools may alter documentation-related work demands, perceived autonomy, professional voice, and the relational aspects of care. From an organizational psychology perspective, AI-assisted clinical documentation can therefore be understood as a work-design and psychosocial job-demand intervention, rather than merely as a technical efficiency tool.

Recent years have seen growing interest in AI-assisted clinical documentation as a potential response to these longstanding pressures (van Buchem et al., 2021; Coiera et al., 2018). These tools include systems used for speech recognition, ambient capture of clinician–patient conversations, automated summarization, note drafting, and structured input within the medical record. Ambient AI scribes, digital scribes, generative AI documentation tools, and large language model (LLM)-supported documentation assistants are now being introduced across a range of healthcare settings (Liu et al., 2024; Palm et al., 2025). Their proposed value lies not only in improving efficiency, but also in reducing the manual and cognitive demands associated with note production (Quiroz et al., 2019). By shifting part of the documentation process away from direct typing and toward AI-supported generation, these tools may alter how healthcare workers experience documentation work during and after patient encounters (Liu et al., 2024; Blackley et al., 2019).

Reported experiences with these technologies, however, are unlikely to be uniformly positive. AI-assisted documentation may reduce documentation burden, lower charting-related stress, improve workflow efficiency, and enhance work satisfaction by allowing healthcare workers to devote more attention to patients and less to clerical tasks (Razaghi et al., 2026; Ma et al., 2025). At the same time, these tools may introduce new sources of strain, including dependence on technology, additional effort related to reviewing and verifying machine-generated text, concern about errors or omissions, a heightened sense of surveillance, and perceived threats to professional autonomy, authorship, or clinical voice (Leung et al., 2025; Fraile Navarro et al., 2025; Bodur et al., 2025). Their psychological consequences are therefore likely to be mixed and shaped by more than technical performance alone. Implementation quality, workflow fit, specialty-specific documentation demands, and clinicians’ perceptions of responsibility and control may shape whether these technologies are experienced as supportive, burdensome, or both (Coiera et al., 2018; Bakken, 2026; Heinrichs et al., 2025).

The evidence base in this area is expanding rapidly, but it remains fragmented and methodologically heterogeneous (Sasseville et al., 2025; Suhail et al., 2026). Studies differ in the types of tools examined, healthcare settings, worker populations, study designs, and outcomes used to represent emotional and occupational experiences. Many evaluations have focused primarily on operational indicators such as documentation time, efficiency, or after-hours charting, whereas emotional outcomes have been reported across a wide range of constructs, including burden, stress, burnout, satisfaction, professional fulfillment, and perceived patient connection (Razaghi et al., 2026; Hodgson and Coiera, 2016; Hodgson et al., 2017). This conceptual and methodological heterogeneity makes it difficult to determine which psychological findings have been most consistently reported, whether reported findings are predominantly favorable, adverse, or mixed, and where important evidence gaps remain. To account for this variability, emotional wellbeing at work in this review is understood in a broad occupational sense, encompassing both distal emotional outcomes and proximal documentation-related psychological experiences that shape how healthcare workers experience clinical work. To avoid treating these heterogeneous outcomes as a single undifferentiated construct, we used a simple proximal–relational–distal conceptual model to organize work-related psychological experiences reported in the literature. In this model, proximal documentation-related experiences refer to outcomes most directly linked to documentation work itself, such as documentation burden, cognitive load, editing burden, frustration, perceived relief, and documentation-related stress. Relational or encounter-level experiences refer to outcomes that reflect how documentation technologies shape the clinician–patient interaction and the meaningful aspects of clinical work, including perceived presence, attentiveness, communication quality, patient connection, and the preservation of clinical voice. Distal occupational outcomes refer to broader work-related consequences, such as burnout, emotional exhaustion, job satisfaction, professional fulfillment, work engagement, work-life balance, turnover intention, and perceived professional autonomy where reported. This model was used as an organizing framework rather than as a presumed causal pathway, because the included studies varied substantially in design, measurement, and follow-up duration.

A scoping review was therefore appropriate for mapping this emerging field. This review identified the types of AI-assisted clinical documentation tools studied, described the characteristics of the included studies, synthesized reported findings related to healthcare workers’ emotional wellbeing at work, and identified priorities for future research. In doing so, it seeks to clarify how AI-assisted clinical documentation has been studied in relation to healthcare workers’ emotional wellbeing at work and under what conditions reported findings appear favorable, adverse, or mixed.

## 2 Methods

This scoping review was conducted in accordance with the Joanna Briggs Institute (JBI) methodological framework (Peters et al., 2020a) and reported in line with the Preferred Reporting Items for Systematic Reviews and Meta-Analyses Extension for Scoping Reviews (PRISMA-ScR) guidelines (Tricco et al., 2018) (Supplementary Table 1). The review protocol was prospectively registered with the Open Science Framework.^1^

### 2.1 Research questions

The review questions were developed to map the existing evidence on AI-assisted clinical documentation in relation to healthcare workers’ emotional wellbeing at work. Specifically, this review addressed the following questions:

1. What types of AI-assisted clinical documentation tools have been examined in the existing literature?
2. What are the main characteristics of the included studies, including healthcare settings, worker populations, and study designs?
3. What favorable, adverse, or mixed findings related to healthcare workers’ emotional wellbeing at work have been reported in studies of AI-assisted clinical documentation?
4. What gaps in the current evidence base should be addressed in future research?

### 2.2 Eligibility criteria

The eligibility criteria for this scoping review were developed in accordance with the Participants, Concept, Context (PCC) framework (Peters et al., 2020a) (Table 1). In addition, types of sources were specified to ensure consistency in study selection.

**Table 1:** Inclusion and exclusion criteria based on the PCC framework.

Category	Inclusion	Exclusion
Population	Healthcare workers engaged in clinical documentation in healthcare settings Physicians Resident physicians and fellows Nurses and nurse practitioners Physician assistants or associates Midwives Psychologists and psychiatrists Allied health professionals Other licensed clinical staff with documentation responsibilities	Patients Caregivers and family members Non-clinical students Purely administrative staff Medical scribes as a separate non-clinical workforce Health informatics developers or information technology staff, unless healthcare worker-specific data could be extracted separately
Concept	AI-assisted clinical documentation tools Tools used for creating, transcribing, summarizing, drafting, structuring, editing, or automating clinical documentation Ambient or digital scribes Natural language processing-based documentation tools Speech recognition systems with AI-enabled functions LLM-supported tools Generative AI systems for note drafting or summarization AI-supported electronic health record documentation tools Outcomes related to healthcare workers’ emotional wellbeing at work	AI applications without a direct documentation function Systems used only for diagnosis, prediction, imaging interpretation, triage, clinical decision support, or risk scoring Tools used only for coding, billing, scheduling, logistics, or administrative automation Traditional dictation or transcription tools without AI components Human scribes without AI assistance Technical algorithm development without application in clinical documentation workflows Technical or operational outcomes without emotional or psychological outcomes for healthcare workers
Context	Real-world healthcare settings Hospitals Outpatient clinics Primary care Emergency departments Intensive care units Specialty clinics Mental health services Telemedicine settings Long-term care settings Community healthcare settings	Simulated settings Laboratory settings Classroom settings Hypothetical settings Non-healthcare workplaces
Types of sources	Original empirical studies with empirical data Quantitative studies Qualitative studies Mixed-methods studies Observational studies Interventional studies Pilot studies Implementation studies Quality improvement studies	Reviews Protocols Editorials Commentaries Letters without original data Conference abstracts without full text Non-peer-reviewed publications

AI, artificial intelligence; LLM, large language model; PCC, Participants, Concept, Context.

#### 2.2.1 Participants

Studies were eligible if they involved healthcare workers engaged in clinical documentation in healthcare settings. Eligible participants included physicians, resident physicians, fellows, nurses, nurse practitioners, physician assistants or associates, midwives, psychologists, psychiatrists, allied health professionals, and other licensed clinical staff with documentation responsibilities. Studies focusing primarily on patients, caregivers, family members, non-clinical students, purely administrative staff, or medical scribes as a separate non-clinical workforce were excluded. Studies involving health informatics developers or information technology staff were also excluded unless data specific to healthcare workers could be extracted separately.

#### 2.2.2 Concept

The concept of interest was the use of AI-assisted clinical documentation tools and their reported relationship with healthcare workers’ emotional wellbeing at work. Eligible studies examined AI-enabled tools that directly supported the creation, transcription, summarization, drafting, structuring, editing, or automation of clinical documentation, including ambient or digital scribes, natural language processing-based documentation tools, speech recognition systems with AI-enabled functions, large language model-supported tools, generative AI systems for note drafting or summarization, and AI-supported electronic health record documentation tools.

To be eligible, studies also had to report outcomes related to healthcare workers’ emotional wellbeing at work. In this review, emotional wellbeing at work was used as an umbrella construct, but it was operationalized through three analytically distinct categories. First, proximal documentation-related experiences included psychological and workflow experiences directly tied to documentation work, such as perceived documentation burden, cognitive load, documentation-related stress or frustration, editing burden, perceived relief, and sense of control during documentation. Second, relational or encounter-level experiences included outcomes reflecting the perceived quality of clinician–patient interaction during AI-assisted documentation, such as presence, attentiveness, communication quality, patient connection, and concerns about clinical voice or authorship. Third, distal occupational outcomes included broader work-related emotional and professional outcomes, such as burnout, emotional exhaustion, job satisfaction, professional fulfillment, work engagement, work-life balance, moral distress, and turnover intention.

This categorization was used to structure eligibility assessment, outcome extraction, and narrative synthesis. Studies were eligible if they reported at least one outcome within these categories, including quantitative measures, qualitative descriptions, or mixed-methods findings related to healthcare workers’ emotional experiences, perceptions, or psychological responses to AI-assisted clinical documentation.

Studies were excluded if they focused on AI applications without a direct documentation function, such as systems used solely for diagnosis, prediction, imaging interpretation, triage, clinical decision support, or risk scoring. Studies of tools used only for coding, billing, scheduling, logistics, or administrative automation were also excluded, as were traditional dictation or transcription tools without AI components, human scribes without AI assistance, and studies limited to technical algorithm development without application in clinical documentation workflows. Studies reporting only technical or operational outcomes, without emotional or psychological outcomes for healthcare workers, were also excluded.

#### 2.2.3 Context

Studies conducted in real-world healthcare settings were eligible, including hospitals, outpatient clinics, primary care, emergency departments, intensive care units, specialty clinics, mental health services, telemedicine settings, and long-term care or community healthcare settings. Studies conducted exclusively in simulated, laboratory, classroom, or hypothetical settings, as well as those in non-healthcare workplaces, were excluded.

#### 2.2.4 Types of sources

This review included original empirical studies, including quantitative, qualitative, and mixed-methods research, as well as observational, interventional, pilot, implementation, and quality improvement studies with empirical data. Reviews, protocols, editorials, commentaries, letters without original data, conference abstracts without full text, and non-peer-reviewed publications were excluded.

### 2.3 Search strategy

In accordance with JBI recommendations (Peters et al., 2020b), the search strategy was developed in consultation with a librarian and the research team and refined through iterative discussions and pilot searches. A systematic search was conducted across five major electronic databases: PubMed, Web of Science, Embase, CINAHL, and PsycINFO. Search strategies were tailored to the indexing systems and search functionalities of each database. Controlled vocabulary terms (e.g., Medical Subject Headings [MeSH] and database-specific subject headings) and free-text terms were combined using Boolean operators (AND, OR, NOT), with truncation and phrase searching applied where appropriate. The core search framework combined terms related to AI-assisted clinical documentation and healthcare workers’ emotional wellbeing at work. The full search strategies for all databases are provided in Supplementary Table 2.

Searches covered each database from inception to the most recent search date. The initial search was completed on January 5, 2026, and subsequently updated on March 16, 2026. To improve the completeness of study identification, the reference lists of included articles were also screened manually for additional relevant records. No language restrictions were applied during the search process. Non-English records were screened using the available English title and abstract when provided, and translation support was used where necessary for eligibility assessment. To ensure methodological rigor and consistency, the search was limited to peer-reviewed academic databases. Consequently, gray literature sources, including preprints, institutional reports, conference materials, and other non-peer-reviewed records, were not systematically searched.

### 2.4 Study selection

All records identified through the search were imported into EndNote X9 for reference management and duplicate removal. Following deduplication, two reviewers (QX and LC) independently screened titles and abstracts according to the predefined eligibility criteria. Full texts of potentially relevant studies were subsequently retrieved and assessed for inclusion. For records without immediately available full text, we attempted retrieval through database links, publisher websites, institutional access, Google Scholar, ResearchGate, and author contact where possible. Disagreements at any stage of the selection process were resolved through discussion with a third reviewer (PW) or through consultation within the research team until consensus was achieved. Inter-reviewer reliability was evaluated using Cohen’s *κ* statistic (McHugh, 2012), which indicated a high level of inter-reviewer agreement at both the title/abstract screening stage (κ = 0.80) and the full-text review stage (κ = 0.82).

### 2.5 Data extraction

Two reviewers (LH and SL) independently extracted data using a structured form developed for this review. The form was pilot-tested and refined before formal extraction to ensure completeness and consistency. Disagreements arising during the extraction process were resolved through discussion and, when necessary, consultation with a third reviewer (NX) until consensus was reached.

The extracted data covered study characteristics, healthcare setting, participant characteristics, AI tool type and function, outcome measures, key findings, and reported outcomes related to healthcare workers’ emotional wellbeing at work. Given the commercial and implementation context of this field, we also extracted reported funding sources, vendor involvement or technology support, institutional or commercial partnerships, author affiliations with vendors, and declared conflicts of interest where available. These items were extracted as reported in the included articles and were not independently verified beyond the published disclosures. The complete extracted dataset from all included studies is provided in Supplementary Table 3. Reported funding sources, vendor involvement, and conflict-of-interest information were extracted as part of the structured data extraction process and are reported in the Results.

### 2.6 Data analysis

Extracted data were analyzed descriptively and synthesized narratively. Descriptive statistics were used to summarize the main characteristics of the included studies, including study design, healthcare setting, participant group, and type of AI-assisted clinical documentation tool, with findings reported as frequencies and percentages where appropriate. Given the heterogeneity of the included evidence, no meta-analysis was performed.

To improve transparency and reproducibility, we applied a predefined coding framework and explicit decision rules to classify both outcome domains and the overall direction of findings for each included study (Table 2). Reported emotional wellbeing outcomes were coded into prespecified domains, including documentation burden, cognitive load/burden, patient connection, burnout, work satisfaction, professional fulfillment/wellbeing, emotional burden/experience, stress/frustration, work-life balance, and turnover/disengagement/exhaustion/control. Conceptually similar outcome labels reported across studies were grouped into broader outcome domains for descriptive synthesis and visualization. A single study could contribute to more than one outcome domain when multiple relevant outcomes were reported. The detailed thematic coding framework is provided in Supplementary Table 4.

**Table 2:** Decision rules for outcome-domain coding and direction-of-findings classification.

Coding component	Decision rule	Use in synthesis
Outcome-domain coding	Reported emotional wellbeing outcomes were assigned to prespecified domains, including documentation burden, cognitive load/burden, patient connection, burnout, work satisfaction, professional fulfillment/wellbeing, stress/frustration, emotional burden/experience, work-life balance, and turnover/disengagement/exhaustion/control.	Used to summarize the distribution of outcome domains and to generate Figure 3.
Multiple domains in one study	A single study could contribute to more than one outcome domain when multiple relevant outcomes were reported.	Domain frequencies reflect the number of studies assessing or describing each domain, not the magnitude of reported findings.
Predominantly favorable	Most reported emotional wellbeing outcomes favored AI-assisted clinical documentation, with no major contradictory or adverse emotional wellbeing findings.	Used for study-level direction coding in Results.
Mixed	Benefits were reported in some domains but not others; key outcomes were null or inconsistent; or favorable findings were accompanied by important concerns such as editing burden, accuracy issues, workflow disruption, usability problems, or concerns about clinical voice, authorship, or autonomy.	Used to retain heterogeneity and implementation trade-offs.
Predominantly adverse	Most emotional wellbeing outcomes were adverse, or the tool introduced substantial new strain without clear reported benefits.	No included study met this criterion.
Unclear	The direction of emotional wellbeing findings could not be determined from the reported data.	Not used in the final classification unless insufficient data were reported.
Study-design weighting	No numerical weighting by study design was applied because this was a scoping review, not an effectiveness review or meta-analysis.	Study design was considered narratively rather than used to weight counts.
Disagreement resolution	Independent coding discrepancies were resolved by discussion and, if needed, adjudication by a third reviewer.	Used to improve coding consistency and reproducibility.

Two reviewers independently coded the reported outcomes and classified the overall direction of findings for each study as predominantly favorable, mixed, predominantly adverse, or unclear. Disagreements in outcome-domain coding or direction-of-findings classification were resolved through discussion; when consensus could not be reached, a third reviewer adjudicated.

For study-level direction coding, a study was classified as predominantly favorable when most reported emotional wellbeing outcomes favored AI-assisted clinical documentation and no major adverse or contradictory findings were reported. A study was classified as mixed when favorable findings were reported in some domains but not others, when findings were inconsistent or null for key emotional wellbeing outcomes, or when reported benefits were accompanied by important concerns such as editing burden, accuracy problems, workflow disruption, reduced usability, or concerns about clinical voice, authorship, or professional autonomy. A study was classified as predominantly adverse when most reported emotional wellbeing outcomes were adverse or when the tool introduced substantial new strain without clear reported benefits. Studies were classified as unclear only when the direction of emotional wellbeing findings could not be determined from the reported data.

During narrative synthesis, we also extracted and contrasted findings related to the redistribution of documentation work. Specifically, we distinguished findings indicating reduced documentation-production burden, such as less typing, shorter note-completion time, lower after-hours charting, reduced recall burden, or lower cognitive task load, from findings indicating increased or persistent AI-management burden, such as reviewing AI-generated drafts, correcting inaccuracies, monitoring omissions, editing style or structure, restoring clinical voice, and deciding when the tool was inappropriate to use. This contrast was used as a descriptive analytic device to explain why proximal documentation outcomes could improve while burnout-related findings remained mixed; it was not treated as a separate quantitative effect estimate.

Because this was a scoping review rather than an effectiveness review or meta-analysis, no numerical weighting by study design was applied in the descriptive counts. Randomized trials, observational studies, pilot studies, and quality improvement evaluations were therefore counted equally for evidence-mapping purposes. However, study design, follow-up duration, setting, and measurement approach were considered in the narrative interpretation and limitations of the evidence.

### 2.7 Critical appraisal of included sources

A formal critical appraisal of the included studies was conducted using the Mixed Methods Appraisal Tool (MMAT) 2018 (Hong et al., 2018). The MMAT was selected because the evidence base included qualitative studies, randomized trials, non-randomized quantitative studies, descriptive quantitative studies, and mixed-methods studies. Each included study was first assessed using the two MMAT screening questions, namely whether the study had clear research questions and whether the collected data allowed the research questions to be addressed. Studies were then appraised using the MMAT criteria corresponding to their methodological category.

Two reviewers independently appraised all included studies. Disagreements were resolved through discussion, and when necessary, consultation with a third reviewer. Appraisal judgments were recorded as “Yes,” “No,” or “Cannot tell.” Because this review was designed as a scoping review rather than an effectiveness review, appraisal results were not used to exclude studies, calculate pooled estimates, or numerically weight findings. Instead, they were used to contextualize the strength, limitations, and interpretability of the evidence base in the narrative synthesis. The complete critical appraisal results are provided in Supplementary Table 5.

## 3 Results

### 3.1 Screening results

**Figure 1:**
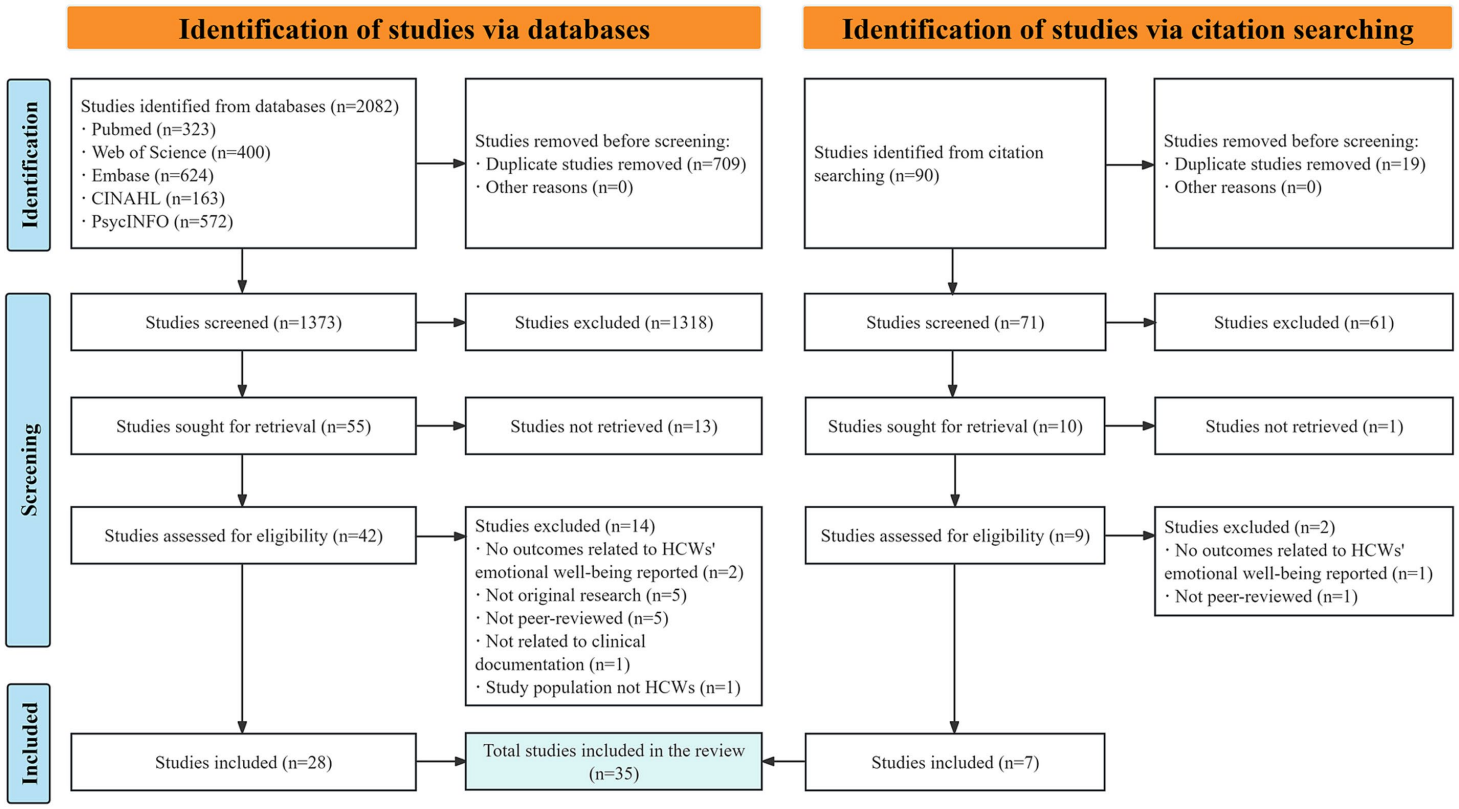
PRISMA flow diagram of the study selection process. Flow diagram showing the identification, screening, eligibility assessment, and inclusion of studies in this scoping review through database searching and citation searching. A total of 2,082 records were identified through database searching and 90 additional records through citation searching. After duplicate removal, screening, and full-text assessment, 35 studies met the inclusion criteria and were included in the review. Reasons for full-text exclusion are presented for both search pathways. HCWs, healthcare workers.

A total of 2,082 records were identified through database searching. After removal of 709 duplicates, 1,373 records underwent title and abstract screening, of which 1,318 were excluded. Forty-two full-text articles were assessed for eligibility after 55 reports were sought for retrieval and 13 could not be retrieved. These 13 records included registry-only records (*n =* 6), conference abstracts without available full text (*n =* 3), one conference paper/proceeding without retrievable full text, and three reports for which full texts remained unavailable despite repeated retrieval attempts and author contact. Fourteen studies were subsequently excluded, leaving 28 studies included from database searching. In addition, 90 records were identified through citation searching. After removal of 19 duplicates and exclusion of 61 records at title and abstract screening, 9 full-text articles were assessed for eligibility, of which 2 were excluded, resulting in 7 additional studies. Overall, 35 studies were included in this scoping review. The study selection process is presented in the PRISMA flow diagram (Figure 1).

### 3.2 Characteristics of included studies

The characteristics of the included studies are summarized in Table 3. The 35 included studies were published between 2023 and 2026 (Misurac et al., 2025; Owens et al., 2024; Shin et al., 2025; Guo et al., 2026; Rabbani et al., 2025; Alpert et al., 2025; Wright et al., 2025; Stults et al., 2025; Schneider et al., 2026; Kuiper et al., 2026; Evans et al., 2025; Bundy et al., 2024; Olson et al., 2025; Chowdhury et al., 2026; Shah et al., 2025b; You et al., 2025; Nguyen et al., 2023; Albrecht et al., 2025; van Linschoten et al., 2026; Van Tiem et al., 2026; Furrukh et al., 2025; Wendt et al., 2025; Pelletier et al., 2025; Galloway et al., 2024; Shah et al., 2025a; Lee et al., 2025; Stults et al., 2026; Omon et al., 2025; Lukac et al., 2025; Duggan et al., 2025; Harvey et al., 2025; Haberle et al., 2024; Marquis et al., 2026; McCrudden et al., 2026; Webb et al., 2026), with most published in 2025 (*n =* 20) (Misurac et al., 2025; Shin et al., 2025; Rabbani et al., 2025; Alpert et al., 2025; Wright et al., 2025; Stults et al., 2025; Evans et al., 2025; Olson et al., 2025; Shah et al., 2025b; You et al., 2025; Albrecht et al., 2025; Furrukh et al., 2025; Wendt et al., 2025; Pelletier et al., 2025; Shah et al., 2025a; Lee et al., 2025; Omon et al., 2025; Lukac et al., 2025; Duggan et al., 2025; Harvey et al., 2025), followed by 2026 (*n =* 10) (Guo et al., 2026; Schneider et al., 2026; Kuiper et al., 2026; Chowdhury et al., 2026; van Linschoten et al., 2026; Van Tiem et al., 2026; Stults et al., 2026; Marquis et al., 2026; McCrudden et al., 2026; Webb et al., 2026), 2024 (*n =* 4) (Owens et al., 2024; Bundy et al., 2024; Galloway et al., 2024; Haberle et al., 2024), and 2023 (*n =* 1) (Nguyen et al., 2023). Most studies were conducted in the United States (*n =* 31) (Misurac et al., 2025; Owens et al., 2024; Shin et al., 2025; Guo et al., 2026; Rabbani et al., 2025; Alpert et al., 2025; Wright et al., 2025; Stults et al., 2025; Schneider et al., 2026; Kuiper et al., 2026; Bundy et al., 2024; Olson et al., 2025; Chowdhury et al., 2026; Shah et al., 2025b; You et al., 2025; Nguyen et al., 2023; Albrecht et al., 2025; Van Tiem et al., 2026; Furrukh et al., 2025; Wendt et al., 2025; Pelletier et al., 2025; Galloway et al., 2024; Shah et al., 2025a; Stults et al., 2026; Lukac et al., 2025; Duggan et al., 2025; Harvey et al., 2025; Haberle et al., 2024; Marquis et al., 2026; McCrudden et al., 2026; Webb et al., 2026), whereas Australia (Evans et al., 2025), the Netherlands (van Linschoten et al., 2026), South Korea (Lee et al., 2025), and Japan (Omon et al., 2025) each contributed one study.

**Table 3:** Characteristics of included studies.

Study ID First author, Year	Country	Study design	Setting	Participants	AI tool (type)	Main outcome domains assessed	Reported funding/commercial context
Misurac et al. (2025)	United States	Before-and-after	Outpatient clinic	Mixed healthcare workers	Nabla (ambient AI scribe)	Burnout/fulfillment; work-life; patient connection	Commercial tool used; funding/vendor role/COI unclear
Owens et al. (2024)	United States	Observational study	Primary care	Mixed healthcare workers	DAX (ambient AI scribe)	Burnout/engagement; emotional burden	Commercial DAX implementation; funding/vendor role/COI unclear
Shin et al. (2025)	United States	Randomized controlled trial	Specialty clinic	Physicians	DAX Copilot (ambient AI scribe)	Burnout; work satisfaction	Commercial DAX Copilot QI trial; prior vendor relationship/training support noted
Guo et al. (2026)	United States	Mixed methods	Mixed settings	Physicians	DAX Copilot/Abridge (ambient AI scribe)	Cognitive/documentation burden; patient connection	No specific grant; no competing interests declared
Rabbani et al. (2025)	United States	Mixed methods	Primary care	Mixed healthcare workers	DAX Copilot (ambient AI scribe)	Cognitive burden; work satisfaction; patient connection	NLM support; Abridge consulting relationship disclosed
Alpert et al. (2025)	United States	Mixed methods	Primary care	Physicians	Commercial AI scribe (ambient AI scribe)	Burnout; cognitive load; patient connection	Commercial AI scribe used; funding/vendor role/COI unclear
Wright et al. (2025)	United States	Prospective observational	Mixed settings	Resident physicians	Abridge (ambient AI scribe)	Cognitive/documentation burden	Commercial Abridge pilot; funding/vendor role/COI unclear
Stults et al. (2025)	United States	Quality improvement	Mixed settings	Mixed healthcare workers	Abridge (ambient AI scribe)	Burnout; cognitive load; work satisfaction; patient connection	Sutter-funded; Sutter–Abridge investment context disclosed
Schneider et al. (2026)	United States	Cross-sectional	Outpatient clinic	Mixed healthcare workers	Ambience Healthcare (ambient AI scribe)	Burnout; cognitive load; work satisfaction; turnover intention	Commercial tool used; no funding/COI declared
Kuiper et al. (2026)	United States	Before-and-after	Telemedicine	Mixed healthcare workers	Ambience AutoScribe (ambient AI scribe)	Burnout; work satisfaction; patient connection	HRSA support; no competing financial interests declared.
Evans et al. (2025)	Australia	Mixed methods	Community healthcare	Allied health professionals	Lyrebird Health (ambient AI scribe)	Documentation burden; job satisfaction; patient connection	No funding; organizational employment context disclosed
Bundy et al. (2024)	United States	Qualitative	Primary care	Physicians	DAX Copilot (ambient AI scribe)	Burnout; cognitive burden; stress; patient connection	Commercial DAX Copilot used; funding/vendor role/COI unclear
Olson et al. (2025)	United States	Quality improvement	Outpatient clinic	Mixed healthcare workers	Abridge (ambient AI scribe)	Burnout; cognitive load; patient connection	Abridge affiliations/relationships disclosed
Chowdhury et al. (2026)	United States	Randomized controlled trial	Outpatient clinic	Physicians	Product A/Product B (ambient AI scribe)	Burnout; work satisfaction	Commercial products compared; no external funding/COI declared
Shah et al. (2025b)	United States	Quality improvement	Mixed settings	Physicians	DAX Copilot (ambient AI scribe)	Burnout; cognitive load	Commercial DAX Copilot pilot; no direct vendor COI clearly reported
You et al. (2025)	United States	Quality improvement	Mixed settings	Mixed healthcare workers	Vendor-unspecified ambient tool (ambient AI scribe)	Burnout/fulfillment; wellbeing; turnover intention	Commercial implementation context; funding/vendor role/COI unclear
Nguyen et al. (2023)	United States	Mixed methods	Specialty clinic	Mixed healthcare workers	Dragon Ambient eXperience (digital scribe)	Burnout; stress	Commercial digital scribe pilot; funding/vendor role/COI unclear
Albrecht et al. (2025)	United States	Quality improvement	Mixed settings	Mixed healthcare workers	Abridge (ambient AI scribe)	Burnout; job satisfaction; documentation burden	Abridge platform; Abridge author affiliation disclosed
van Linschoten et al. (2026)	Netherlands	Mixed methods	Primary care	Physicians	Ambient scribe (ambient AI scribe)	Documentation burden; work satisfaction; patient connection	External funding and implementation collaboration disclosed
Van Tiem et al. (2026)	United States	Qualitative	Mixed settings	Mixed healthcare workers	Nabla (ambient AI scribe)	Job satisfaction; patient connection	Institutional support; no competing interests declared
Furrukh et al. (2025)	United States	Pilot	Specialty clinic	Resident physicians	Dragon Ambient eXperience (ambient AI scribe)	Burnout; cognitive load; work satisfaction; patient connection	DAX pilot; funding/vendor role/COI unclear
Wendt et al. (2025)	United States	Randomized controlled trial	Primary care	Physicians	Dragon Ambient eXperience (ambient AI scribe)	Burnout; frustration; documentation burden; patient connection	DAX licenses provided by Nuance/Microsoft; no COI declared
Pelletier et al. (2025)	United States	Pilot	Specialty clinic	Mixed healthcare workers	Abridge (digital scribe)	Burnout; cognitive load; work satisfaction; work-life	Abridge BAA/vendor data context; funding/COI unclear
Galloway et al. (2024)	United States	Before-and-after	Mixed settings	Mixed healthcare workers	Ambient listening solution (ambient AI scribe)	Wellbeing; patient connection	Commercial ambient solution; funding/vendor role/COI unclear
Shah et al. (2025a)	United States	Qualitative	Mixed settings	Physicians	DAX Copilot (ambient AI scribe)	Cognitive load; job satisfaction; work-life; patient connection	Commercial DAX Copilot pilot; funding/vendor role/COI unclear
Lee et al. (2025)	South Korea	Before-and-after	Emergency department	Physicians	Y-KNOT-EDN (LLM-supported documentation tool)	Cognitive load; frustration	Internal LLM documentation system; commercial COI unclear
Stults et al. (2026)	United States	Qualitative	Mixed settings	Mixed healthcare workers	Abridge (ambient AI scribe)	Cognitive burden; work satisfaction; patient connection	Abridge pilot context; funding/vendor role/COI unclear
Omon et al. (2025)	Japan	Before-and-after	Community healthcare	Allied health professionals	medimo (generative AI documentation tool)	Cognitive load; frustration	Pleap/medimo development contribution noted
Lukac et al. (2025)	United States	Randomized controlled trial	Outpatient clinic	Physicians	DAX Copilot/Nabla (ambient AI scribe)	Burnout; stress; cognitive load; work satisfaction	UCLA-funded; DAX Copilot and Nabla compared
Duggan et al. (2025)	United States	Quality improvement	Outpatient clinic	Mixed healthcare workers	DAX Copilot (ambient AI scribe)	Documentation/cognitive burden; patient connection; work-life	Commercial DAX Copilot QI study; funding/vendor role/COI unclear
Harvey et al. (2025)	United States	Pilot	Specialty clinic	Physicians	Ambient AI scribe (ambient AI scribe)	Burnout; cognitive load; patient connection	Commercial ambient AI pilot; funding/vendor role/COI unclear
Haberle et al. (2024)	United States	Cohort	Outpatient clinic	Mixed healthcare workers	Dragon Ambient eXperience (ambient AI scribe)	Work engagement; wellbeing; work-life	Nuance DAX used; no funding/COI declared
Marquis et al. (2026)	United States	Mixed methods	Emergency department	Physicians	DAX Copilot (ambient AI scribe)	Work satisfaction; documentation burden	Commercial DAX Copilot ED pilot; funding/vendor role/COI unclear
McCrudden et al. (2026)	United States	Retrospective observational mixed methods	Telemedicine	Mixed healthcare workers	Smart Notes (generative AI documentation tool)	Documentation burden; cognitive load; work satisfaction	Talkspace-developed tool; all authors employed by Talkspace
Webb et al. (2026)	United States	Randomized controlled trial	Emergency department	Physicians	DAX/Abridge (ambient AI scribe)	Documentation burden; cognitive load; work satisfaction	DAX/Abridge compared; vendor training/support noted

AI, artificial intelligence; LLM, large language model.

A range of study designs was represented. Mixed-methods studies (*n =* 8) (Guo et al., 2026; Rabbani et al., 2025; Alpert et al., 2025; Evans et al., 2025; Nguyen et al., 2023; van Linschoten et al., 2026; Marquis et al., 2026; McCrudden et al., 2026) were most common, followed by quality improvement studies (*n =* 6) (Stults et al., 2025; Olson et al., 2025; Shah et al., 2025b; You et al., 2025; Albrecht et al., 2025; Duggan et al., 2025), before-and-after studies (*n =* 5) (Misurac et al., 2025; Kuiper et al., 2026; Galloway et al., 2024; Lee et al., 2025; Omon et al., 2025), and randomized controlled trials (*n =* 5) (Shin et al., 2025; Chowdhury et al., 2026; Wendt et al., 2025; Lukac et al., 2025; Webb et al., 2026). Other designs included qualitative studies (*n =* 4) (Bundy et al., 2024; Van Tiem et al., 2026; Shah et al., 2025a; Stults et al., 2026), pilot studies (*n =* 3) (Furrukh et al., 2025; Pelletier et al., 2025; Harvey et al., 2025), one cross-sectional study (Schneider et al., 2026), one observational study (Owens et al., 2024), one prospective observational study (Alpert et al., 2025), and one cohort study (Haberle et al., 2024).

The studies were conducted across multiple healthcare settings. Mixed clinical settings (*n =* 10) (Guo et al., 2026; Wright et al., 2025; Stults et al., 2025; Shah et al., 2025b; You et al., 2025; Albrecht et al., 2025; Van Tiem et al., 2026; Galloway et al., 2024; Shah et al., 2025a; Stults et al., 2026) were most frequently reported, followed by outpatient clinics (*n =* 7) (Misurac et al., 2025; Schneider et al., 2026; Olson et al., 2025; Chowdhury et al., 2026; Lukac et al., 2025; Duggan et al., 2025; Haberle et al., 2024), primary care (*n =* 6) (Owens et al., 2024; Rabbani et al., 2025; Alpert et al., 2025; Bundy et al., 2024; van Linschoten et al., 2026; Wendt et al., 2025), and specialty clinics (*n =* 5) (Shin et al., 2025; Nguyen et al., 2023; Furrukh et al., 2025; Pelletier et al., 2025; Harvey et al., 2025). Smaller numbers of studies were conducted in emergency departments (*n =* 3) (Lee et al., 2025; Marquis et al., 2026; Webb et al., 2026), telemedicine settings (*n =* 2) (Kuiper et al., 2026; McCrudden et al., 2026), and community healthcare settings (*n =* 2) (Evans et al., 2025; Omon et al., 2025). In terms of participant populations, mixed healthcare worker samples (*n =* 17) (Misurac et al., 2025; Owens et al., 2024; Rabbani et al., 2025; Stults et al., 2025; Schneider et al., 2026; Kuiper et al., 2026; Olson et al., 2025; You et al., 2025; Nguyen et al., 2023; Albrecht et al., 2025; Van Tiem et al., 2026; Pelletier et al., 2025; Galloway et al., 2024; Stults et al., 2026; Duggan et al., 2025; Haberle et al., 2024; McCrudden et al., 2026) and physicians (*n =* 14) (Shin et al., 2025; Guo et al., 2026; Alpert et al., 2025; Bundy et al., 2024; Chowdhury et al., 2026; Shah et al., 2025b; van Linschoten et al., 2026; Wendt et al., 2025; Shah et al., 2025a; Lee et al., 2025; Lukac et al., 2025; Harvey et al., 2025; Marquis et al., 2026; Webb et al., 2026) were most commonly included, whereas resident physicians (*n =* 2) (Wright et al., 2025; Furrukh et al., 2025) and allied health professionals (*n =* 2) (Evans et al., 2025; Omon et al., 2025) were less frequently represented. Regarding specialty, mixed specialties (*n =* 22) (Misurac et al., 2025; Guo et al., 2026; Alpert et al., 2025; Wright et al., 2025; Stults et al., 2025; Schneider et al., 2026; Kuiper et al., 2026; Evans et al., 2025; Olson et al., 2025; Chowdhury et al., 2026; Shah et al., 2025b; You et al., 2025; Nguyen et al., 2023; Albrecht et al., 2025; Van Tiem et al., 2026; Galloway et al., 2024; Shah et al., 2025a; Stults et al., 2026; Omon et al., 2025; Lukac et al., 2025; Duggan et al., 2025; Haberle et al., 2024) predominated, followed by primary care (*n =* 4) (Owens et al., 2024; Bundy et al., 2024; van Linschoten et al., 2026; Wendt et al., 2025), pediatrics (*n =* 3) (Shin et al., 2025; Rabbani et al., 2025; Pelletier et al., 2025), emergency medicine (*n =* 3) (Lee et al., 2025; Marquis et al., 2026; Webb et al., 2026), surgery (*n =* 2) (Furrukh et al., 2025; Harvey et al., 2025) and mental health (*n =* 1) (McCrudden et al., 2026).

Reported funding sources, vendor involvement, and declared conflicts of interest are summarized in Table 3 and Supplementary Table 6. Across the included studies, the commercial and implementation context of AI-assisted documentation was frequently relevant. Most included studies evaluated commercially available or institutionally deployed AI documentation products, including DAX/DAX Copilot (Owens et al., 2024; Shin et al., 2025; Shah et al., 2025b; Wendt et al., 2025), Abridge (Guo et al., 2026; Stults et al., 2025; Olson et al., 2025; Albrecht et al., 2025; Pelletier et al., 2025), Nabla (Misurac et al., 2025; Chowdhury et al., 2026; Lukac et al., 2025), Ambience Healthcare (Schneider et al., 2026; Kuiper et al., 2026), Lyrebird Health (Evans et al., 2025), and related ambient or digital scribe platforms. Several studies were conducted as institutional pilots, quality improvement evaluations, or health-system implementation studies in which commercial tools were deployed under local implementation arrangements (Shin et al., 2025; Stults et al., 2025; Olson et al., 2025; Shah et al., 2025b; Albrecht et al., 2025; Wendt et al., 2025; Duggan et al., 2025). Some studies reported no specific external funding or no declared competing interests, whereas others disclosed vendor-provided technology, vendor-supported implementation or training, institutional investment relationships, author affiliations with vendors, consulting relationships, or employment by the organization that developed or deployed the tool (Rabbani et al., 2025; Stults et al., 2025; Evans et al., 2025; Olson et al., 2025; Albrecht et al., 2025; van Linschoten et al., 2026; Wendt et al., 2025; McCrudden et al., 2026). Because disclosure practices varied across studies, the absence of a declared conflict of interest should not be interpreted as evidence that no commercial or implementation influence was present.

### 3.3 Critical appraisal of included studies

All 35 included studies met the two MMAT screening criteria and were therefore included in the full critical appraisal. Based on MMAT methodological categories, five studies were appraised as randomized controlled trials, 17 as quantitative non-randomized studies, one as a quantitative descriptive study, four as qualitative studies, and eight as mixed-methods studies. Overall, four studies were judged to have few methodological concerns, 21 had some concerns, and 10 had substantial concerns (Supplementary Table 5).

The qualitative studies generally showed stronger internal coherence, with clear alignment between research questions, interview-based data collection, thematic analysis, and interpretation. However, their transferability was limited by single-organization settings, pilot-user samples, or specific implementation contexts. By contrast, most quantitative and mixed-methods studies had some or substantial methodological concerns. Common limitations included non-randomized or uncontrolled designs, short follow-up periods, small or self-selected samples, reliance on self-reported outcomes, incomplete or unclear response data, variable uptake of the AI documentation tools, and limited adjustment for confounding.

Among the randomized studies, methodological strengths included random allocation, crossover or controlled designs, and, in some cases, objective electronic health record metrics. However, these studies were generally open-label, often short in duration, and sometimes affected by limited intervention uptake, carryover or order effects, or incomplete survey-based outcomes. For the non-randomized studies, the main concerns were the frequent use of single-group pre-post designs, limited control for secular trends or implementation support, and the difficulty of separating tool effects from early-adopter enthusiasm, novelty effects, or local workflow changes. These appraisal findings indicate that the current evidence base is useful for mapping reported experiences and implementation signals, but remains less definitive for causal claims about burnout or broader occupational wellbeing.

### 3.4 Types and functions of AI-assisted clinical documentation tools

**Figure 2:**
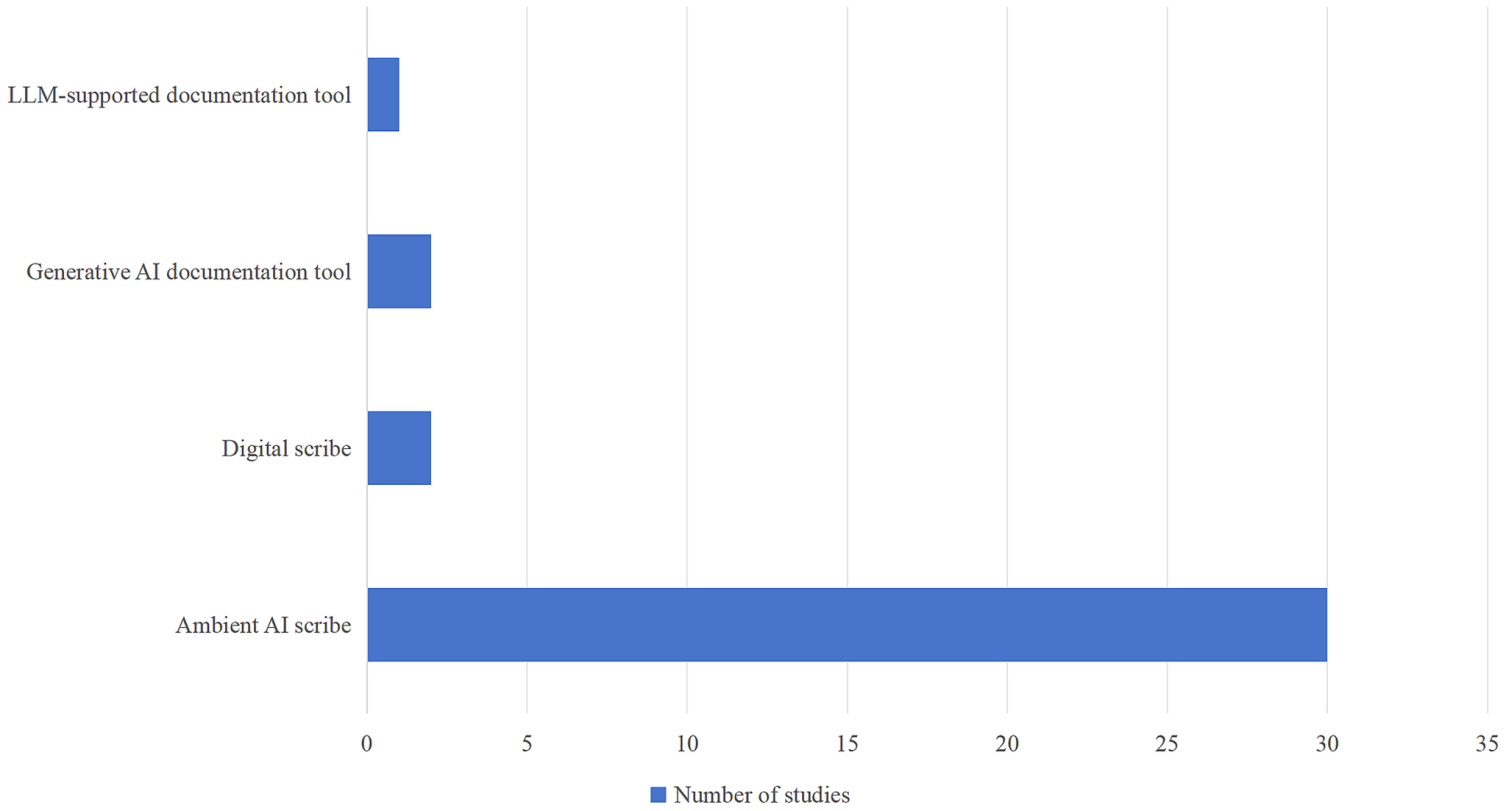
Types of AI-assisted clinical documentation tools examined in the included studies. Distribution of AI-assisted clinical documentation tool types across the 35 included studies. Ambient AI scribe tools were the most frequently examined (*n =* 30), followed by digital scribes (*n =* 2), generative AI documentation tools (*n =* 2), and LLM-supported documentation tools (*n =* 1). The figure highlights the marked predominance of ambient AI scribe systems in the current evidence base. AI, artificial intelligence; LLM, large language model.

The types of AI-assisted clinical documentation tools examined in the included studies are shown in Figure 2. Most studies evaluated ambient AI scribe tools (*n =* 30) (Misurac et al., 2025; Owens et al., 2024; Shin et al., 2025; Guo et al., 2026; Rabbani et al., 2025; Alpert et al., 2025; Wright et al., 2025; Stults et al., 2025; Schneider et al., 2026; Kuiper et al., 2026; Evans et al., 2025; Bundy et al., 2024; Olson et al., 2025; Chowdhury et al., 2026; Shah et al., 2025b; You et al., 2025; Albrecht et al., 2025; van Linschoten et al., 2026; Van Tiem et al., 2026; Furrukh et al., 2025; Wendt et al., 2025; Galloway et al., 2024; Shah et al., 2025a; Stults et al., 2026; Lukac et al., 2025; Duggan et al., 2025; Harvey et al., 2025; Haberle et al., 2024; Marquis et al., 2026; Webb et al., 2026). Other tool types included digital scribes (*n =* 2) (Nguyen et al., 2023; Pelletier et al., 2025), generative AI documentation tools (*n =* 2) (Omon et al., 2025; McCrudden et al., 2026), and LLM-supported documentation tools (*n =* 1) (Lee et al., 2025).

Across the included studies, the main reported functions of these tools were transcription, summarization, clinical note drafting, structuring of documentation content, and documentation support within the electronic health record. Many studies described tools that generated draft notes from clinician–patient conversations for subsequent clinician review and editing. Frequently reported products included Abridge, DAX/DAX Copilot, and Nabla, while several studies evaluated vendor-unspecified AI-assisted documentation systems.

### 3.5 Reported findings related to healthcare workers’ emotional wellbeing at work

**Figure 3:**
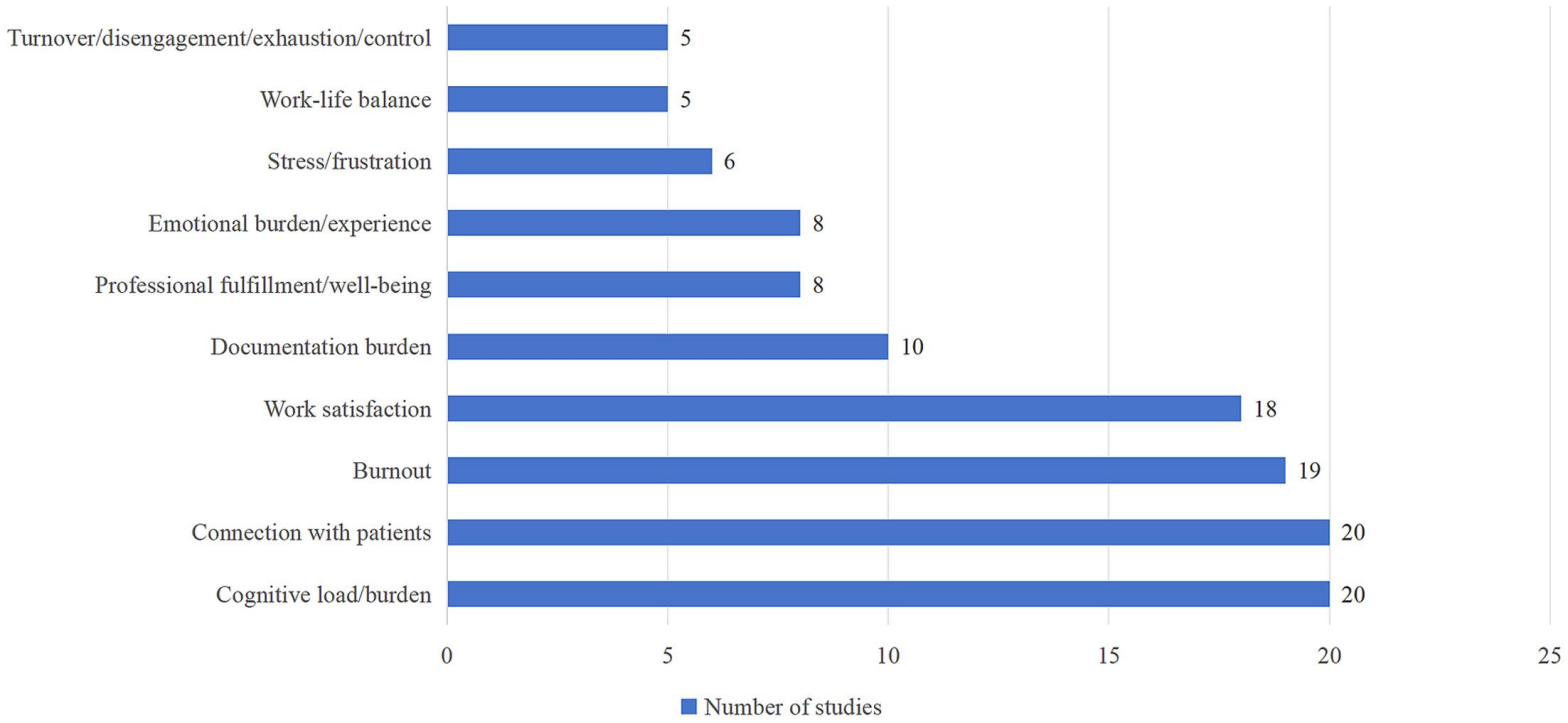
Emotional wellbeing outcome domains assessed in the included studies. Frequency of emotional wellbeing outcome domains reported across the included studies. For visualization, conceptually similar outcome labels were grouped into broader domains. Cognitive load/burden and patient connection were the most frequently assessed domains (*n =* 20 each), followed by burnout (*n =* 19), work satisfaction (*n =* 18), documentation burden (*n =* 10), professional fulfillment/wellbeing (*n =* 8), emotional burden/experience (*n =* 8), stress/frustration (*n =* 6), work-life balance (*n =* 5), and turnover/disengagement/exhaustion/control (*n =* 5). Frequencies represent the number of studies assessing each domain rather than the magnitude or direction of reported findings.

The emotional wellbeing outcome domains assessed across the included studies are presented in Figure 3, in which related outcome labels were grouped into broader domains for visualization. The reported findings and their overall directions are summarized in Table 4. Based on the predefined coding framework and direction-of-findings criteria presented in Table 2, 23 of the 35 included studies were classified as reporting predominantly favorable findings (Misurac et al., 2025; Guo et al., 2026; Wright et al., 2025; Stults et al., 2025; Schneider et al., 2026; Kuiper et al., 2026; Evans et al., 2025; Bundy et al., 2024; Olson et al., 2025; Chowdhury et al., 2026; Shah et al., 2025b; You et al., 2025; Albrecht et al., 2025; Furrukh et al., 2025; Wendt et al., 2025; Pelletier et al., 2025; Galloway et al., 2024; Lee et al., 2025; Lukac et al., 2025; Duggan et al., 2025; Harvey et al., 2025; McCrudden et al., 2026; Webb et al., 2026), and 12 were classified as reporting mixed findings (Owens et al., 2024; Shin et al., 2025; Rabbani et al., 2025; Alpert et al., 2025; Nguyen et al., 2023; van Linschoten et al., 2026; Van Tiem et al., 2026; Shah et al., 2025a; Stults et al., 2026; Omon et al., 2025; Haberle et al., 2024; Marquis et al., 2026). Commonly reported favorable findings included perceived or measured reductions in documentation burden and cognitive load/burden, as well as reported improvements in work satisfaction, professional fulfillment/wellbeing, and patient connection. Several studies also reported reduced after-hours documentation and more favorable workflow experiences during clinical encounters. Mixed findings were reported in studies in which improvements were observed in some domains but not others, or in which reported benefits were accompanied by concerns related to implementation and use. A common pattern within these mixed findings was discordance between favorable clinician-reported experiences, such as improved satisfaction, reduced perceived burden, or better patient connection, and less consistent changes in objective workflow metrics, such as after-hours documentation, pajama time, note completion time, or other electronic health record-based measures, as summarized in Tables 4, 5.

**Table 4:** Summary of reported findings on AI-assisted clinical documentation in relation to healthcare workers’ emotional wellbeing at work.

Outcome domain	Overall direction of findings	Summary of reported findings
Patient connection	Predominantly favorable	Studies frequently reported improved ability to focus on patients during encounters, greater perceived presence, and enhanced clinician–patient connection.
Burnout	Mixed	Several studies reported reductions in burnout or burnout-related dimensions, whereas others found improvement in some subdomains only or no clear change in overall burnout measures.
Cognitive load/burden	Predominantly favorable	Reduced mental demand, cognitive burden, and documentation-related workload were commonly reported across studies.
Work satisfaction	Predominantly favorable	Many studies described improved work satisfaction, more favorable documentation experiences, and greater perceived ease of clinical work.
Documentation burden	Predominantly favorable	Studies commonly reported reduced documentation burden, including reduced effort associated with note completion and lower after-hours charting burden.
Professional fulfillment/wellbeing	Mixed	Some studies reported improved professional fulfillment, work engagement, or broader wellbeing at work, although findings were not uniform across studies.
Work-life balance	Predominantly favorable	A subset of studies reported better work-life balance or integration, often in relation to reduced documentation burden and lower after-hours work.
Stress / frustration	Mixed	Some studies described reductions in stress or frustration, whereas others reported persistent frustration related to editing needs, note accuracy, or workflow disruption.
Emotional burden / experience	Mixed	Qualitative and mixed-methods studies described relief from documentation burden in some cases, alongside concerns related to trust, usability, and fit within clinical practice.
Turnover/disengagement/exhaustion/control	Mixed	A small number of studies assessed turnover intention, interpersonal disengagement, or work exhaustion, with variable findings across settings and tools.

AI, artificial intelligence.

**Table 5:** Result-level contrast between reduced documentation-production burden and increased AI-management burden.

Area of documentation work	Reported reduction in documentation-production burden	Reported increase or persistence of AI-management burden	Interpretation for burnout and wellbeing
Note drafting and typing	Several studies reported shorter documentation time, reduced time in notes, fewer manual documentation tasks, or easier note completion (Guo et al., 2026; Stults et al., 2025; Olson et al., 2025; Wendt et al., 2025; Pelletier et al., 2025; Lukac et al., 2025; Duggan et al., 2025; McCrudden et al., 2026).	Other studies described the need to review, edit, correct, or sometimes substantially restructure AI-generated drafts (Rabbani et al., 2025; Bundy et al., 2024; Nguyen et al., 2023; Van Tiem et al., 2026; Marquis et al., 2026).	AI may reduce the burden of producing a first draft, but clinicians remain responsible for verifying and finalizing the note.
Cognitive and attentional burden	Studies frequently reported reduced cognitive load, less split attention, or greater ability to focus on patients during encounters (Rabbani et al., 2025; Evans et al., 2025; van Linschoten et al., 2026; Shah et al., 2025a; Duggan et al., 2025).	Some studies reported persistent vigilance related to omissions, inaccuracies, hallucinated or misheard content, and uncertainty about note completeness (Rabbani et al., 2025; van Linschoten et al., 2026; Van Tiem et al., 2026; Stults et al., 2026; Marquis et al., 2026; Webb et al., 2026).	Real-time cognitive burden may decrease, but supervisory vigilance may increase during or after note review.
After-hours documentation and work spillover	Some studies reported reduced after-hours documentation, pajama time, or work-home spillover (Stults et al., 2025; Olson et al., 2025; Pelletier et al., 2025; Duggan et al., 2025).	Other studies reported variable or limited improvement in objective workflow metrics, or benefits that depended on uptake, workflow fit, and editing requirements (Owens et al., 2024; Nguyen et al., 2023; Wendt et al., 2025; Haberle et al., 2024).	Work-life gains may occur when editing burden is low, but may be attenuated when review and correction tasks remain substantial.
Note quality, structure, and clinical voice	AI-generated drafts were often described as useful starting points that reduced recall burden and supported documentation completion (Bundy et al., 2024; Pelletier et al., 2025; McCrudden et al., 2026).	Concerns were reported about note length, formatting, style, specialty-specific fit, loss of clinical voice, authorship, and professional control (Rabbani et al., 2025; van Linschoten et al., 2026; Van Tiem et al., 2026; Stults et al., 2026).	Documentation work may shift from composing text to restoring accuracy, style, and professional authorship.
Workflow fit and use-case boundaries	Several studies reported favorable experiences when tools fit the encounter type, specialty workflow, or documentation task (Guo et al., 2026; Shah et al., 2025b; Lukac et al., 2025; Duggan et al., 2025).	Barriers included device access, language limitations, specialty-specific documentation needs, and decisions about when not to use the tool (Kuiper et al., 2026; Shah et al., 2025a; Marquis et al., 2026; Webb et al., 2026).	Benefits depend on implementation fit; poor fit may convert time savings into new workflow-management demands.
Burnout-related interpretation	Proximal outcomes such as documentation burden and cognitive load were more consistently favorable (Table 4).	Burnout, stress/frustration, emotional burden, and broader wellbeing outcomes remained mixed (Table 4).	Reducing note-production work does not necessarily translate into broad burnout reduction when supervisory and organizational burdens persist.

This table provides a descriptive result-level contrast rather than a quantitative effect estimate. Study design and methodological concerns are further contextualized in Supplementary Table 5.

### 3.6 Redistribution of documentation work

Across the included studies, AI-assisted clinical documentation appeared to reduce the production side of documentation work more consistently than it eliminated documentation burden overall. As summarized in Table 5, reported benefits commonly involved reduced typing, faster note drafting, lower documentation effort, reduced after-hours charting, and less cognitive splitting between patient interaction and documentation (Guo et al., 2026; Stults et al., 2025; Olson et al., 2025; Wendt et al., 2025; Pelletier et al., 2025; Lukac et al., 2025; Duggan et al., 2025; McCrudden et al., 2026). However, several studies also described new or persistent management tasks, including reviewing AI-generated drafts, correcting inaccuracies, monitoring omissions, editing note structure or style, preserving clinical voice, and deciding when the tool was not appropriate for specific encounters or documentation contexts (Rabbani et al., 2025; Bundy et al., 2024; Nguyen et al., 2023; Van Tiem et al., 2026; Stults et al., 2026; Marquis et al., 2026; Webb et al., 2026).

This redistribution pattern helps explain why documentation burden and cognitive load were often reported as improved, whereas burnout-related findings remained mixed (Tables 4, 5). In other words, AI-assisted documentation may reduce the burden of producing notes, but some of this burden may shift to supervisory, verification, and workflow-management work. The emotional experience associated with these tools may therefore depend on whether the reduction in typing and recall burden is greater than the added burden of supervising machine-generated documentation.

### 3.7 Distribution of evidence across populations, tools, and outcome domains

The evidence base was concentrated in specific populations, settings, and tool types. Most studies were conducted in the United States and involved physicians or mixed clinician groups. Ambient AI scribes dominated the literature, whereas digital scribes, generative AI documentation tools, and LLM-supported documentation tools were less frequently examined. Several studies were pilot or quality improvement evaluations. Outcome domains and directions of findings also varied across studies, with patient connection, cognitive load/burden, burnout, and work satisfaction assessed most often. Other aspects of emotional wellbeing at work were reported less frequently, and outcome assessment methods varied across validated instruments, study-specific questionnaires, and qualitative reports.

## 4 Discussion

### 4.1 Principal findings

This scoping review shows that the literature on AI-assisted clinical documentation has expanded quickly, but remains methodologically limited and conceptually heterogeneous. Across the 35 included studies, the most consistent findings concerned proximal and relational outcomes, including lower documentation burden, reduced cognitive load, better work satisfaction, and improved perceived patient connection, rather than uniform reductions in burnout. By contrast, findings for burnout, professional fulfillment, stress, and broader emotional wellbeing were more variable. This pattern is consistent with Table 4, where the clearest favorable signals clustered around documentation burden, cognitive burden, work satisfaction, and patient connection, while other outcome domains were less consistent.

Interpreted through the proximal–relational–distal model, the evidence was strongest for proximal documentation-related experiences and relational encounter-level experiences. Improvements were most consistently reported for documentation burden, cognitive load, perceived relief, work satisfaction with documentation processes, and patient connection. In contrast, distal occupational outcomes, particularly burnout and broader emotional wellbeing, were more heterogeneous. This pattern is consistent with the interpretation that AI-assisted documentation may first be reflected in the immediate psychological experience of documentation work and the perceived quality of clinical encounters, whereas broader occupational outcomes are likely to depend on additional organizational and contextual factors.

A second key finding is the marked concentration of evidence around ambient AI scribes. Most included studies evaluated ambient systems, whereas only a small minority examined digital scribes, generative AI documentation tools, or LLM-supported documentation platforms. In practical terms, then, this review reflects a field shaped largely by one technological and workflow model rather than a balanced evidence base across multiple forms of documentation AI (Guo et al., 2026; Stults et al., 2025; Wendt et al., 2025; Lukac et al., 2025). Accordingly, the findings of this review should be understood primarily as evidence about ambient AI scribe implementation in predominantly United States healthcare settings, rather than as evidence about all AI-assisted clinical documentation tools across healthcare systems.

Overall, the evidence supports a more restrained interpretation than is sometimes implied in discussions of this technology. The evidence suggests that AI-assisted documentation has been associated with perceived reductions in several documentation-related strains that matter to healthcare workers. It does not, however, support the stronger claim that these tools offer a general solution to occupational distress. At this stage, a more defensible interpretation is narrower: these systems may be associated with favorable changes in selected proximal drivers of emotional strain, while broader occupational findings appear dependent on clinical context, workflow fit, specialty, and implementation quality (Guo et al., 2026; Stults et al., 2025; Wendt et al., 2025; Lukac et al., 2025).

### 4.2 Interpretation of findings

Across the included studies, the value of AI-assisted documentation appears to extend beyond efficiency alone. Many studies have reported less charting effort and shorter documentation time. Even so, the more meaningful contribution may lie in how these tools are associated with changes in the experience of clinical work itself. Healthcare workers often described feeling more present during encounters, less cognitively split between listening and typing, and more able to focus on the patient rather than the screen (Kuiper et al., 2026; Evans et al., 2025; Van Tiem et al., 2026; Duggan et al., 2025).

This distinction is important because documentation burden is not only a matter of time; it can fragment attention and disrupt the cognitive work required for clinical practice (Moy et al., 2021; Moy et al., 2023; Ratanawongsa et al., 2017). Ambient systems are especially relevant here because they act at the point where documentation and patient interaction most directly compete. Rather than simply speeding up note production after the visit, they may reduce the attentional tension embedded within the encounter itself.

This may help explain why several studies reported better patient connection or encounter quality even when objective electronic health record metrics changed only modestly (Rabbani et al., 2025; van Linschoten et al., 2026; Stults et al., 2026). It may also explain why perceived gains in satisfaction and reduced burden sometimes appeared before consistent changes in pajama time, throughput, or other operational indicators (Rabbani et al., 2025; Chowdhury et al., 2026; Shah et al., 2025b; Pelletier et al., 2025). This pattern suggests that favorable emotional or relational experiences may be reported relatively early, particularly in relation to lower perceived cognitive task load and improved attention to patients, whereas operational gains may be less immediate and may depend on better workflow integration over time (van Buchem et al., 2021; Liu et al., 2024; Rabbani et al., 2025).

The mixed findings were therefore not simply inconsistent results across unrelated studies. Rather, they often reflected a specific pattern of discordance between subjective and objective outcomes. Several studies reported favorable clinician-perceived changes, including reduced documentation burden, improved satisfaction, greater perceived presence during encounters, or better patient connection, even when objective workflow indicators changed modestly, inconsistently, or not at all (Rabbani et al., 2025; Chowdhury et al., 2026; Shah et al., 2025b; van Linschoten et al., 2026; Pelletier et al., 2025; Stults et al., 2026). Metrics such as after-hours documentation, pajama time, note closure, documentation time, or electronic health record activity may not capture all aspects of how clinicians experience documentation work (Rabbani et al., 2025; Chowdhury et al., 2026; Shah et al., 2025b; Pelletier et al., 2025). Conversely, subjective improvements may partly reflect perceived relief, novelty effects, early-adopter enthusiasm, or better encounter experience before measurable workflow efficiencies become apparent. This discordance suggests that AI-assisted documentation may first be reflected in clinicians’ perceived documentation experience and patient interaction, whereas objective operational benefits may require more sustained use, better integration, and adaptation of clinical workflows.

The allied health, telemedicine, and qualitative studies are particularly informative on this point. They suggest that the emotional value of AI-assisted documentation may be greatest where documentation visibly intrudes on therapeutic communication and relationship-building (Kuiper et al., 2026; Evans et al., 2025; Stults et al., 2026). From this perspective, the relevance of AI-assisted documentation extends beyond workload reduction; it may also help preserve aspects of clinical practice that clinicians associate with meaning, connection, and professional identity (Melnick et al., 2020; Coiera et al., 2018).

### 4.3 Burnout and related outcomes

Burnout was one of the most commonly assessed outcomes in the included studies, but findings were among the most inconsistent. Several studies reported reductions in burnout or in related dimensions such as disengagement and work exhaustion (Misurac et al., 2025; Schneider et al., 2026; Wendt et al., 2025; Lukac et al., 2025). Others found improvement only in selected components, only among more frequent users, or only in subjective perceptions rather than broader composite measures (Owens et al., 2024; Shin et al., 2025; Rabbani et al., 2025; Chowdhury et al., 2026). Some studies showed clear gains in documentation burden or cognitive load without a parallel change in global burnout (Nguyen et al., 2023; Pelletier et al., 2025).

This mixed pattern likely reflects more than inconsistency in study findings. Burnout is a distal, multidetermined construct. Documentation burden is certainly one contributor, but occupational distress is shaped by multiple interacting factors, including workload intensity, staffing adequacy, professional autonomy, organizational culture, and sources of moral distress (Meredith et al., 2022; Rotenstein et al., 2025; Burns et al., 2021; Brender et al., 2025). It is therefore not surprising that an intervention targeting documentation may improve proximal sources of strain without consistently shifting broader burnout scores, especially in short-term pilot studies.

Interpretation is further complicated by the heterogeneity of outcome measurement. Across the included studies, burnout-related outcomes were assessed using different instruments and adjacent constructs, including single-item burnout measures, exhaustion, disengagement, professional fulfillment, work-life strain, and broader wellbeing indices (Misurac et al., 2025; Owens et al., 2024; Furrukh et al., 2025; Lukac et al., 2025). These constructs overlap, but they are not interchangeable. That limits direct comparison and makes it difficult to treat burnout as a single, stable outcome category across the literature.

Accordingly, the strongest conclusion at present is not that AI-assisted documentation reliably reduces burnout in a general sense. It is, rather, that these tools appear to reduce documentation-proximal burdens which may, under some conditions, contribute to lower burnout. This more modest interpretation better reflects what the current evidence can support.

### 4.4 Distribution of evidence across tool types

The predominance of ambient AI scribes is itself a substantive finding. Most included studies examined ambient systems, while evidence on digital scribes, generative note-drafting tools, and LLM-assisted documentation remains comparatively sparse. As a result, many of the favorable signals identified in this review reflect one technological and workflow model rather than AI-assisted clinical documentation as a whole.

There are understandable reasons for this concentration. Ambient scribes address one of the most visible pain points in contemporary clinical work: the need to attend to the patient while simultaneously producing documentation. By capturing and structuring the encounter in real time, these systems aim to reduce both recall burden and keyboard burden. More bounded tools, such as discharge-note assistants or *post hoc* summarization systems, may improve narrower components of documentation work, but they do not intervene as directly in the real-time tension between documentation and patient interaction (Lee et al., 2025; Omon et al., 2025; McCrudden et al., 2026).

At the same time, the smaller body of evidence on non-ambient systems remains important because it suggests that documentation AI should not be treated as a single intervention. The LLM-supported emergency discharge-note study and the generative AI studies in rehabilitation and mental health suggest that more bounded tools can also reduce workload and improve acceptability in specific contexts (Lee et al., 2025; Omon et al., 2025; McCrudden et al., 2026). Even so, these studies also indicate that outcomes depend heavily on task structure, specialty-specific note requirements, and the extent to which generated output aligns with actual clinical workflow.

This point is reinforced by comparative and randomized studies, which suggest that product-level differences in usability, perceived quality, and documentation outcomes are not trivial (Chowdhury et al., 2026; Lukac et al., 2025; Duggan et al., 2025). Ambient AI scribes should therefore be treated as a heterogeneous product category rather than a uniform intervention. Future syntheses and implementation decisions will need to specify more clearly which systems, which use cases, and which workflow assumptions are actually under evaluation.

### 4.5 Implementation challenges, autonomy, and trade-offs

These implementation challenges are also relevant to how clinicians experience control, responsibility, and authorship in documentation work. As shown in Table 5, a recurring theme across the included studies is that documentation burden is often reduced, but not simply removed. Instead, some of that burden appears to be redistributed into other kinds of work: reviewing drafts, correcting inaccuracies, restructuring notes, monitoring omissions, editing style, and deciding when the tool is inappropriate to use (Rabbani et al., 2025; Bundy et al., 2024; Nguyen et al., 2023; Van Tiem et al., 2026; Marquis et al., 2026). This redistribution may help explain why proximal outcomes such as documentation burden and cognitive load were often favorable, whereas burnout-related findings remained mixed. In particular, mixed findings may arise when AI tools reduce the perceived burden of producing documentation but introduce new supervisory tasks, such as reviewing AI-generated drafts, correcting inaccuracies, monitoring omissions, editing note structure or style, preserving clinical voice, or deciding when the tool is inappropriate to use, which may not be fully captured by either subjective satisfaction scores or objective electronic health record metrics (Rabbani et al., 2025; Bundy et al., 2024; Nguyen et al., 2023; Van Tiem et al., 2026; Stults et al., 2026; Marquis et al., 2026; Webb et al., 2026).

This redistribution matters not only in practical terms, but also in how the burden of documentation is understood. A tool may reduce typing while increasing the vigilance required to supervise machine-generated text. Several studies raised concerns about note length, specialty-specific fit, speaker attribution, hallucinated or omitted content, and the handling of nuance or sensitive information (Rabbani et al., 2025; van Linschoten et al., 2026; Van Tiem et al., 2026; Stults et al., 2026). These concerns were particularly visible in communication-rich or structurally distinctive settings, such as pediatrics and mental health, where note quality depends on subtleties that generic ambient generation may not capture well (Rabbani et al., 2025; Pelletier et al., 2025; McCrudden et al., 2026).

The qualitative literature is especially helpful here. Clinical notes are not merely clerical outputs; they also function as professional documents that encode reasoning, accountability, communication style, and clinical judgment (Van Tiem et al., 2026). When clinicians expressed discomfort with AI-generated note voice or structure, they were not simply reacting to stylistic variation. They were pointing to a deeper tension between automation and authorship. In that sense, the success of AI-assisted documentation depends not only on whether it saves time, but also on whether clinicians regard the output as clinically trustworthy, communicatively appropriate, and sufficiently aligned with their own documentation style and professional judgment (Fraile Navarro et al., 2025; Hodgson et al., 2017).

For that reason, favorable findings should be interpreted with some caution. The current evidence suggests favorable changes, but these changes are often conditional on editing burden remaining acceptable. Once supervisory burden becomes too high, the technology may shift work rather than substantially reduce it. This trade-off is likely to vary across specialties, note types, and individual documentation styles.

### 4.6 Heterogeneity across settings and populations

The heterogeneity of the evidence base is not merely a methodological inconvenience; it is one of the substantive findings of this review. The included studies varied substantially in setting, professional group, specialty, tool type, implementation maturity, and outcome measurement. The literature was also concentrated in the United States and weighted toward outpatient and mixed clinical settings, with physicians and mixed clinician groups predominating. Allied health, trainees, mental health, emergency medicine, and contexts outside the United States remain comparatively underrepresented (Wright et al., 2025; Evans et al., 2025; Lee et al., 2025; McCrudden et al., 2026; Webb et al., 2026).

This unevenness has important implications. It suggests that average findings may conceal real variation in who benefits, why they benefit, and under what conditions. Some healthcare workers may experience substantial relief because the technology fits their encounter structure and documentation practices. Others may derive only modest value if editing demands offset drafting gains or if the system is poorly aligned with specialty-specific documentation requirements (Rabbani et al., 2025; Van Tiem et al., 2026; Stults et al., 2026). The pediatric studies, for example, show both considerable promise and clear limitations (Shin et al., 2025; Rabbani et al., 2025; Pelletier et al., 2025). The resident and trainee studies suggest potential benefit, but they also raise unresolved questions about supervision, learning, and the development of documentation competence (Wright et al., 2025; Furrukh et al., 2025). The mental health and allied health literature broadens the field in useful ways, yet remains too limited to support strong generalization (Evans et al., 2025; Omon et al., 2025; McCrudden et al., 2026).

This heterogeneity also points to a more specific research agenda. Rather than continuing to ask whether AI-assisted documentation is beneficial in general, future studies should examine which tools work, for whom, in which tasks, under what implementation conditions, and with what trade-offs (Scipion et al., 2025; Kanaparthy et al., 2026).

### 4.7 Limitations of the included evidence

The MMAT-based critical appraisal provides important context for interpreting the findings of this review. Although the included studies consistently suggest that AI-assisted clinical documentation may reduce documentation-proximal strain, the methodological strength of the evidence remains uneven. Most studies had some or substantial methodological concerns, particularly among quantitative and mixed-methods evaluations. These concerns do not invalidate the reported benefits, but they limit the certainty with which improvements can be attributed to AI documentation tools themselves. These patterns are detailed in Supplementary Table 5 and were used to calibrate the interpretation of the narrative synthesis.

Several limitations were recurrent across the evidence base. Many studies were short-term, single-site, uncontrolled, and reliant on self-reported outcomes. Randomized studies were relatively few. Even when objective data were included, outcomes were often limited to narrow electronic health record proxies such as time in notes, note closure, or after-hours activity, without consistent integration of note quality, adoption intensity, patient experience, or longer-term occupational outcomes. These limitations matter directly for interpretation. Novelty effects, early-adopter enthusiasm, enhanced implementation support, and local workflow changes cannot be ruled out. Causal inference also remains limited because improvements observed during pilot periods may partly reflect selective participation by clinicians more inclined to adopt new technologies.

The appraisal also highlights a mismatch between the strength of claims and the maturity of the evidence. Evidence is strongest for feasibility, acceptability, perceived documentation relief, and short-term reductions in documentation-related cognitive burden. It is weaker for durable changes in burnout, professional fulfillment, work-life balance, turnover intention, and long-term emotional wellbeing. This distinction is important because documentation-related burden is only one contributor to broader occupational distress. Therefore, short-term reductions in documentation burden should not be interpreted as definitive evidence that AI documentation tools reduce burnout as a multidimensional occupational outcome.

The commercial and implementation context of this literature also warrants caution. A substantial proportion of included studies evaluated commercially available AI documentation products within active institutional pilots, quality improvement programs, or health-system implementation partnerships. In several cases, studies involved vendor-provided tools, usage data, training, implementation support, institutional investment relationships, author affiliations with vendors, consulting relationships, or employment by the organization that developed or deployed the tool. These relationships do not invalidate the findings, and many studies transparently disclosed no competing interests or no specific external funding. However, in a rapidly expanding commercial market for AI documentation systems, favorable implementation signals may be more likely to be evaluated, written up, and published than neutral or unfavorable experiences, creating a potential risk of publication and implementation bias. This commercial and publication context may partly contribute to the predominance of favorable direction-of-findings classifications in the current review. Therefore, the reported favorable findings should be interpreted alongside the disclosed funding, vendor involvement, and conflict-of-interest information, as well as alongside the MMAT-based methodological appraisal.

A further evidence gap is the relative scarcity of patient-reported outcomes, despite frequent claims that AI-assisted documentation improves clinician–patient interaction and patient-centered care (Evans et al., 2025; van Linschoten et al., 2026). Potential harms were also rarely studied with the same rigor as benefits. Although inaccuracies, omissions, note-quality concerns, and editing burden were acknowledged in several studies, these were usually treated as secondary rather than primary outcomes (Van Tiem et al., 2026; Stults et al., 2026; Lukac et al., 2025; Webb et al., 2026). As a result, the field still lacks robust evidence on the safety, longer-term behavioral consequences, and professional implications of routine AI-assisted note generation.

At present, the literature is stronger on feasibility and acceptability than on durable effectiveness, safety, and longer-term professional consequences. Future primary studies should therefore prioritize longer follow-up, multicenter pragmatic designs, more rigorous assessment of note quality, clearer reporting of implementation context, and stronger linkage between subjective experience and objective workflow data. From an organizational psychology perspective, these tools should be evaluated as work-design interventions that redistribute cognitive, relational, and supervisory demands, rather than simply as documentation-efficiency technologies.

### 4.8 Strengths and limitations of this review

This review has several strengths. It addresses a timely and fast-moving topic, applies a clear PCC-based eligibility framework, and focuses specifically on healthcare workers’ emotional wellbeing at work rather than on technical performance or efficiency alone. It also captures a broad range of empirical study designs and clinical settings, allowing a more nuanced picture of how documentation AI is being experienced in real-world practice.

Several limitations of this review should also be acknowledged. As a scoping review, this study was designed to map the evidence base rather than to estimate pooled effect sizes or establish causal efficacy. Its purpose was breadth and conceptual synthesis, not quantitative effect estimation. Although a formal MMAT-based critical appraisal was conducted, the appraisal results were used to contextualize the evidence rather than to exclude studies, weight findings, or generate graded certainty ratings. Therefore, the findings should be interpreted as a structured evidence map with methodological calibration, rather than as a meta-analytic or certainty-graded assessment of intervention effectiveness.

Because study designs were not numerically weighted, the direction-of-findings categories should be interpreted as descriptive classifications rather than as indicators of comparative evidentiary strength or causal certainty. For example, a large uncontrolled implementation study and a small randomized trial were both counted as individual studies in the descriptive synthesis, even though their inferential strength differed. The MMAT appraisal was therefore used to help readers interpret the maturity and limitations of the evidence base alongside the direction of reported findings. We also extracted funding sources, vendor involvement, and declared conflicts of interest as reported in the included studies. However, these disclosures were not independently verifiable, and reporting practices varied across journals and study types. Therefore, this review may undercapture informal vendor influence, unpublished implementation agreements, or commercial pressures that were not explicitly disclosed.

The search covered multiple major peer-reviewed databases and included citation searching, but gray literature was not systematically searched or included. In a rapidly evolving field such as clinical AI documentation, this may have excluded some of the most up-to-date implementation experiences, including preprints, institutional reports, conference materials, and other emerging real-world evidence. As a result, the review may underrepresent the newest implementation cases and the earliest signals of practice change.

Finally, because the available literature was concentrated in the United States and focused predominantly on ambient AI scribes, the generalizability of this review across international settings and across the broader landscape of documentation AI remains limited.

### 4.9 Implications for research and practice

AI-assisted documentation should not be implemented or evaluated primarily as a productivity technology, nor should its success be judged only by short-term changes in burnout scores. A more realistic and evidence-aligned expectation is that these tools may first reduce documentation-proximal burdens, such as cognitive load, documentation burden, and attentional fragmentation, which may only under some conditions translate into broader reductions in burnout over time. If health systems implement these tools primarily to increase throughput, they may risk undermining one of the benefits that healthcare workers appear to value most, namely relief from the cognitive and emotional burden of documentation. Several studies have already pointed to this tension, with clinicians expressing concern that time saved may simply be converted into pressure to see more patients rather than into better working conditions (Bundy et al., 2024; Van Tiem et al., 2026).

Implementation should therefore focus on fit, governance, and longitudinal monitoring rather than adoption alone. Tool selection, specialty adaptation, note-template flexibility, training, and mechanisms for rapid user feedback are all likely to influence whether the technology genuinely reduces burden or merely shifts it elsewhere. Evaluation should also move beyond electronic health record efficiency measures to include editing burden, note quality, clinician trust, patient experience, and longer-term wellbeing outcomes. This distinction between proximal strain reduction and broader burnout outcomes is important for both interpretation and implementation, because it suggests that the value of documentation AI may lie less in functioning as a standalone solution to occupational distress and more in being associated with reduced specific sources of strain within documentation workflows.

For research, the key question is not simply whether favorable findings are reported, but under what conditions, for whom, and with what trade-offs such findings emerge. More multicenter and longer-duration studies are needed, alongside comparative evaluations across products, specialties, and documentation tasks. Outcome frameworks should better integrate psychometric, behavioral, and documentation-quality measures. Potential harms should also be studied as seriously as benefits, including inaccuracies, overreliance, note-quality degradation, and potential downstream effects on training, reasoning, and professional communication (Van Tiem et al., 2026; Stults et al., 2026; Webb et al., 2026). Without such evidence, the field risks scaling a promising but still incompletely understood intervention.

## 5 Conclusion

In conclusion, this scoping review found that AI-assisted clinical documentation was most consistently associated with clinician-reported relief in documentation-proximal experiences, particularly perceived documentation burden and cognitive load, with possible favorable findings for work satisfaction and patient connection. However, the current evidence base remains narrow: it is concentrated largely in United States healthcare settings and dominated by studies of ambient AI scribe systems. Therefore, the findings should not be generalized uncritically to other healthcare systems, professional groups, specialties, or AI documentation technologies.

Evidence for broader emotional wellbeing outcomes, including burnout and related occupational outcomes, remains limited and mixed. The observed mixed findings also suggest that subjective improvements in documentation experience may not always align with objective workflow metrics. Given the methodological limitations of many included studies, these findings should be interpreted as reported associations and perceived changes rather than causal evidence of improved emotional wellbeing. Future research should prioritize rigorous, multicenter, longitudinal evaluations using validated wellbeing measures, objective workflow metrics, and transparent assessment of implementation benefits and harms.

## Data Availability

The original contributions presented in the study are included in the article/Supplementary material, further inquiries can be directed to the corresponding author.

## Footnotes

1
https://osf.io/m8e9v
