# Supplementary material for: Evidence on artificial intelligence-assisted clinical documentation and healthcare workers’ emotional wellbeing at work: a scoping review
Source: Front Psychol. 2026 Jun 24;17:1840884. doi: 10.3389/fpsyg.2026.1840884 (PMC13341515; doi:10.3389/fpsyg.2026.1840884)
Supplement: Supplementary file 2 [file Table_2.DOCX]

**Supplementary Table 2:** Search Strategy.

| **Database** | **Search terms** | **Results** |
| --- | --- | --- |
| PubMed | ( "Artificial Intelligence"[Mesh] OR "Natural Language Processing"[Mesh] OR "Speech Recognition Software"[Mesh] OR artificial intelligence[tiab] OR AI[tiab] OR "natural language processing"[tiab] OR NLP[tiab] OR "speech recognition"[tiab] OR "voice recognition"[tiab] OR dictation[tiab] OR "large language model*"[tiab] OR LLM[tiab] OR "generative AI"[tiab] OR ChatGPT[tiab] OR "ambient scribe*"[tiab] OR "digital scribe*"[tiab] OR "AI scribe*"[tiab] OR scribe*[tiab] OR automation[tiab] OR "automated"[tiab] ) AND ( "Medical Records"[Mesh] OR "Clinical Documentation"[tiab] OR "medical documentation"[tiab] OR "clinical note*"[tiab] OR "progress note*"[tiab] OR "SOAP note*"[tiab] OR "documentation burden"[tiab] OR "note burden"[tiab] OR "electronic health record*"[tiab] OR EHR[tiab] OR EMR[tiab] ) AND ( "Health Personnel"[Mesh] OR "Physicians"[Mesh] OR "Nurses"[Mesh] OR "healthcare worker*"[tiab] OR "health care worker*"[tiab] OR clinician*[tiab] OR physician*[tiab] OR doctor*[tiab] OR nurse*[tiab] OR "medical resident*"[tiab] OR resident*[tiab] OR "physician assistant*"[tiab] OR "nurse practitioner*"[tiab] ) AND ( "Burnout, Professional"[Mesh] OR burnout[tiab] OR "emotional exhaustion"[tiab] OR "Job Satisfaction"[Mesh] OR "job satisfaction"[tiab] OR "work satisfaction"[tiab] OR "Stress, Psychological"[Mesh] OR stress[tiab] OR "work stress"[tiab] OR "occupational stress"[tiab] OR "Well-Being"[Mesh] OR wellbeing[tiab] OR "well-being"[tiab] OR "psychological well-being"[tiab] OR "work engagement"[tiab] OR "employee engagement"[tiab] OR "turnover intention"[tiab] OR "intention to leave"[tiab] OR "quality of life"[Mesh] OR "quality of life"[tiab] ) | 323 |
| Web of Science | TS=(( "artificial intelligence" OR "AI" OR "generative AI" OR "LLM" OR "natural language processing" OR "ambient" OR "automated" OR "digital scribe" ) AND ( "clinical documentation" OR "medical record*" OR "electronic health record*" OR "EHR" OR "EMR" OR "charting" OR "note-writing" ) AND ( "healthcare worker*" OR "clinician*" OR "physician*" OR "doctor*" OR "nurse*" OR "medical student*" OR "intern*" OR "resident*" OR "trainee*" ) AND ( "emotion*" OR "well-being" OR "burnout" OR "stress" OR "satisfaction" OR "anxiety" OR "frustration" OR "mental health" OR "psychological" OR "cognitive load" )) | 400 |
| Embase | ('artificial intelligence'/exp OR 'natural language processing'/exp OR 'speech recognition software'/exp OR 'large language model*': ti, ab OR LLM: ti, ab OR 'generative AI': ti, ab OR ChatGPT: ti, ab OR 'ambient scribe*': ti, ab OR 'digital scribe*': ti, ab OR 'AI scribe*': ti, ab OR scribe*: ti, ab) AND ('medical records'/exp OR 'clinical documentation': ti, ab OR 'medical documentation': ti, ab OR 'clinical note*': ti, ab OR 'progress note*': ti, ab OR 'SOAP note*': ti, ab OR 'electronic health record*': ti, ab OR EHR: ti, ab OR EMR: ti, ab)  AND ('health personnel'/exp OR 'physician'/exp OR 'nurse'/exp OR 'healthcare worker*': ti, ab OR 'health care worker*': ti, ab OR clinician*: ti, ab OR physician*: ti, ab OR doctor*: ti, ab OR nurse*: ti, ab OR 'medical resident*': ti, ab OR resident*: ti, ab OR 'physician assistant*': ti, ab OR 'nurse practitioner*': ti, ab) AND ('burnout'/exp OR burnout: ti, ab OR 'emotional exhaustion': ti, ab OR 'job satisfaction'/exp OR 'job satisfaction': ti, ab OR 'work satisfaction': ti, ab OR 'psychological stress'/exp OR stress: ti, ab OR 'occupational stress': ti, ab OR 'work stress': ti, ab OR 'wellbeing'/exp OR wellbeing: ti, ab OR 'well-being': ti, ab OR 'psychological well-being': ti, ab OR 'work engagement': ti, ab OR 'employee engagement': ti, ab OR 'turnover intention': ti, ab OR 'intention to leave': ti, ab OR 'quality of life'/exp OR 'quality of life': ti, ab) | 624 |
| CINAHL | (TI ( ("artificial intelligence" OR AI OR "generative AI" OR "large language model*" OR LLM* OR "machine learning" OR "natural language processing" OR NLP OR "speech recognition" OR "voice recognition" OR "ambient scribe*" OR "AI scribe*" OR "digital scribe*") N3 (documentation OR "clinical documentation" OR charting OR "clinical note*" OR note* OR "medical record*" OR "electronic health record*" OR EHR* OR EMR* OR dictation OR transcription)) OR AB (("artificial intelligence" OR AI OR "generative AI" OR "large language model*" OR LLM* OR "machine learning" OR "natural language processing" OR NLP OR "speech recognition" OR "voice recognition" OR "ambient scribe*" OR "AI scribe*" OR "digital scribe*") N3 (documentation OR "clinical documentation" OR charting OR "clinical note*" OR note* OR "medical record*" OR "electronic health record*" OR EHR* OR EMR* OR dictation OR transcription)) OR (((MH "Artificial Intelligence+") OR (MH "Natural Language Processing") OR (MH "Machine Learning") OR (MH "Speech Recognition Software")) AND ((MH "Medical Records") OR (MH "Documentation") OR (MH "Nursing Records")))) AND ((MH "Health Personnel+") OR (MH "Nurses+") OR (MH "Physicians+") OR (MH "Allied Health Personnel") OR TI (clinician* OR "healthcare worker*" OR "health care worker*" OR provider* OR physician* OR doctor* OR nurse* OR "nurse practitioner*" OR "medical staff" OR "healthcare professional*" OR "health care professional*") OR AB (clinician* OR "healthcare worker*" OR "health care worker*" OR provider* OR physician* OR doctor* OR nurse* OR "nurse practitioner*" OR "medical staff" OR "healthcare professional*" OR "health care professional*") ) AND ((MH "Burnout, Professional") OR (MH "Occupational Stress") OR (MH "Job Satisfaction") OR (MH "Emotions") OR (MH "Mental Health") OR TI ("emotional well-being" OR wellbeing OR "well-being" OR burnout OR stress OR distress OR fatigue OR workload OR "mental workload" OR "job satisfaction" OR morale OR "emotional exhaustion") OR AB ("emotional well-being" OR wellbeing OR "well-being" OR burnout OR stress OR distress OR fatigue OR workload OR "mental workload" OR "job satisfaction" OR morale OR "emotional exhaustion") ) | 163 |
| PsycINFO | ( ( (artificial intelligence or generative AI or large language model* or LLM* or machine learning or natural language processing or NLP or speech recognition or voice recognition or ambient scribe* or AI scribe* or digital scribe*) adj3 (documentation or clinical documentation or charting or clinical note* or note* or medical record* or electronic health record* or EHR* or EMR* or dictation or transcription) ).ti, ab. or ( (exp artificial intelligence/ or exp machine learning/ or exp natural language processing/) and (documentation or clinical documentation or charting or clinical note* or note* or medical record* or electronic health record* or EHR* or EMR* or dictation or transcription).ti, ab. )) and ( exp health personnel/ or exp physicians/ or exp nurses/ or (clinician* or healthcare worker* or health care worker* or provider* or physician* or doctor* or nurse* or nurse practitioner* or medical staff or healthcare professional* or health care professional*).ti, ab.) and ( exp occupational stress/ or exp burnout/ or exp job satisfaction/ or exp emotional states/ or exp psychological well-being/ or (emotional well-being or wellbeing or well-being or burnout or stress or distress or fatigue or workload or mental workload or cognitive burden or job satisfaction or morale or emotional exhaustion or occupational well-being or technostress). ti, ab.) | 572 |
