# Supplementary material for: Evidence on artificial intelligence-assisted clinical documentation and healthcare workers’ emotional wellbeing at work: a scoping review
Source: Front Psychol. 2026 Jun 24;17:1840884. doi: 10.3389/fpsyg.2026.1840884 (PMC13341515; doi:10.3389/fpsyg.2026.1840884)
Supplement: Supplementary file 4 [file Table_4.DOCX]

**Supplementary Material 4:** Higher-order conceptual grouping and detailed thematic coding framework for outcome categorization and narrative synthesis.

**1. Higher-order conceptual grouping of emotional well-being outcomes**

To improve conceptual clarity, emotional well-being at work was treated as an umbrella construct and further organized into three higher-order categories: proximal documentation-related experiences, relational or encounter-level experiences, and distal occupational outcomes. This higher-order grouping was used to support narrative synthesis and to distinguish outcomes most directly linked to documentation work from broader occupational consequences.

| **Higher-order category** | **Core meaning** | **Outcome domains included** |
| --- | --- | --- |
| Proximal documentation-related experiences | Outcomes most directly related to documentation work and AI-assisted note generation | Documentation burden; cognitive load/burden; stress/frustration; emotional burden/experience; work-life balance related to after-hours documentation |
| Relational or encounter-level experiences | Outcomes reflecting perceived changes in clinician–patient interaction during documentation | Patient connection; presence; attentiveness; communication quality; clinical voice or authorship concerns |
| Distal occupational outcomes | Broader work-related emotional or professional outcomes influenced by multiple organizational factors | Burnout; emotional exhaustion; work satisfaction; professional fulfillment/well-being; work engagement; turnover intention; disengagement; moral distress |

**2. Detailed coding framework for outcome domains**

The domains below were used to categorize reported emotional well-being outcomes and related themes across the included studies.

| **Higher-order category** | **Outcome domain** | **Operational definition** | **Illustrative labels or examples** | **Coding notes** |
| --- | --- | --- | --- | --- |
| Proximal documentation-related experiences | Cognitive load/burden | Perceived mental effort, attentional strain, task switching, or documentation-related cognitive burden during or after clinical work. | cognitive load; mental burden; reduced split attention; easier note completion | Use when findings primarily describe changes in mental effort or cognitive demands. |
| Relational or encounter-level experiences | Patient connection | Perceived presence, attentiveness, communication quality, rapport, or relational engagement during patient encounters. | better eye contact; improved listening; greater presence; improved patient interaction | Use when outcomes relate to clinician-patient connection rather than general satisfaction. |
| Distal occupational outcomes | Burnout | Burnout or closely related constructs, including emotional exhaustion, disengagement, and work exhaustion, when explicitly reported as such. | burnout; exhaustion; disengagement; work exhaustion | Use only when burnout-related constructs are directly assessed or clearly described. |
| Distal occupational outcomes | Work satisfaction | Satisfaction with work, workflow, documentation experience, or overall job-related experience. | job satisfaction; workflow satisfaction; satisfaction with documentation process | Use for satisfaction-related outcomes. When satisfaction referred specifically to documentation workflow, this was noted in the narrative synthesis. Do not merge with professional fulfillment unless explicitly framed that way. |
| Proximal documentation-related experiences | Documentation burden | Perceived burden, effort, or strain associated specifically with documentation tasks. | documentation burden; charting burden; clerical burden; reduced after-hours charting burden | Use for documentation-specific burden rather than general stress or burnout. |
| Distal occupational outcomes | Professional fulfillment/well-being | Favorable occupational well-being, meaning, fulfillment, or sense of professional effectiveness. | professional fulfillment; occupational well-being; sense of meaning or relief | Apply when studies describe broader favorable work-related well-being beyond simple satisfaction. |
| Proximal documentation-related experiences | Emotional burden/experience | Subjective emotional responses to documentation work or AI tool use, including perceived relief or emotional strain. | emotional burden; emotional relief; emotional experience of documentation | Use when studies foreground emotional responses related to documentation or AI-assisted documentation without mapping them to a narrower domain. |
| Proximal documentation-related experiences | Stress/frustration | Stress, frustration, anxiety, or tension related to documentation or AI-supported workflows. | stress; frustration; anxiety; tension about note quality or omissions | Code separately from burnout when reported as a more proximal reaction. |
| Proximal documentation-related experiences | Work-life balance | Reported changes in work-life integration, time outside work, or spillover of documentation into personal time. | work-life balance; pajama time; after-hours work; work-home interference | Use when outcomes relate to time boundaries and spillover into non-work life, particularly when linked to after-hours documentation. |
| Distal occupational outcomes | Turnover/disengagement/exhaustion/control | Related occupational constructs that were infrequently reported and grouped for descriptive visualization. | turnover intention; sense of control; disengagement; exhaustion | Grouped for visualization because these constructs were reported less frequently and heterogeneously. |

**3. Coding principles used in the review**

- Conceptually similar outcome labels were grouped into broader domains for descriptive synthesis and visualization.
- A single study could contribute to more than one outcome domain when multiple emotional well-being outcomes were reported.
- Domain frequencies represent the number of studies assessing or describing a domain, not the magnitude or direction of the reported finding.
- Narrative findings on perceived benefits, concerns, implementation challenges, and workflow fit were reviewed alongside coded outcome domains.
- Mixed findings were retained when studies reported both beneficial and challenging aspects of AI-assisted clinical documentation.
- Outcome domains were additionally mapped onto three higher-order categories: proximal documentation-related experiences, relational or encounter-level experiences, and distal occupational outcomes.
- The higher-order categories were used for conceptual organization and narrative interpretation rather than as mutually exclusive causal stages.

**4. Note on interpretation**

This framework was developed to support consistent grouping of heterogeneous outcomes across quantitative, qualitative, and mixed-methods studies. It was used as an analytic aid for outcome categorization, conceptual organization, and narrative comparison rather than for quantitative pooling or causal inference.

The higher-order grouping was intended to clarify whether reported outcomes were concentrated in proximal documentation-related experiences, relational or encounter-level experiences, or broader distal occupational outcomes. Because the included studies varied substantially in design, measurement, and follow-up duration, the proximal–relational–distal structure should be interpreted as an organizing framework rather than as a tested causal pathway.

**5. Decision rules for study-level direction-of-findings classification**

In addition to outcome-domain coding, each included study was assigned an overall direction-of-findings classification. This classification was based on reported emotional well-being outcomes rather than technical performance alone. Operational or efficiency outcomes were considered only when they were explicitly linked to emotional well-being, documentation burden, cognitive load, work-life balance, or clinician experience.

Predominantly favorable findings were coded when most reported emotional well-being outcomes favored AI-assisted clinical documentation and when no major contradictory, null, or adverse findings were reported for key emotional well-being outcomes.

Mixed findings were coded when studies reported favorable findings in some emotional well-being domains but null, inconsistent, or adverse findings in others; when improvements were limited to selected subdomains; or when benefits were accompanied by substantial implementation concerns, including editing burden, note accuracy concerns, workflow disruption, usability problems, loss of clinical voice, authorship concerns, or perceived threats to professional autonomy.

Predominantly adverse findings were coded when most emotional well-being outcomes were adverse or when AI-assisted documentation introduced substantial new emotional, cognitive, or workflow-related strain without clear reported benefit. No included study met this criterion.

Unclear findings were reserved for studies in which the emotional well-being direction could not be determined from the reported data. No study was included in the main favorable/mixed summary unless sufficient information was available to determine direction.

No numerical weighting by study design was applied. Study design was extracted and reported to contextualize the strength and maturity of the evidence, but randomized trials, observational studies, pilot studies, and quality improvement studies were counted equally in descriptive mapping. Differences in study design, follow-up duration, setting, and measurement approach were considered in the narrative synthesis and limitations rather than in a weighted quantitative analysis.
