# Supplementary material for: Evidence on artificial intelligence-assisted clinical documentation and healthcare workers’ emotional wellbeing at work: a scoping review
Source: Front Psychol. 2026 Jun 24;17:1840884. doi: 10.3389/fpsyg.2026.1840884 (PMC13341515; doi:10.3389/fpsyg.2026.1840884)
Supplement: Supplementary file 5 [file Table_5.DOCX]

**Supplementary Table 5. Critical appraisal of included studies using the Mixed Methods Appraisal Tool (MMAT) 2018**

Notes: All included studies passed the two MMAT screening questions: S1, clear research questions; S2, collected data allow the research questions to be addressed. Ratings: Y, yes; N, no; CT, cannot tell; NA, not applicable. The overall interpretation was used to contextualize the narrative synthesis and was not used to exclude studies, weight findings, or generate pooled effect estimates.

Overall interpretation categories: Few concerns = most applicable MMAT criteria met with no major threats to the credibility of the study findings; Some concerns = one or more limitations that may affect interpretation but do not invalidate the study; Substantial concerns = multiple or major limitations that substantially weaken confidence in effect-related conclusions.

**Summary of appraisal results**

| **MMAT category** | **Number of studies** | **Few concerns** | **Some concerns** | **Substantial concerns** | **Total** |
| --- | --- | --- | --- | --- | --- |
| Quantitative randomized controlled trials | 5 | 0 | 5 | 0 | 5 |
| Quantitative non-randomized studies | 17 | 0 | 7 | 10 | 17 |
| Quantitative descriptive studies | 1 | 0 | 1 | 0 | 1 |
| Qualitative research | 4 | 4 | 0 | 0 | 4 |
| Mixed-methods studies | 8 | 0 | 8 | 0 | 8 |
| **All included studies** | **35** | **4** | **21** | **10** | **35** |

**Quantitative randomized controlled trials (n = 5)**

RCT1, randomization appropriately performed; RCT2, groups comparable at baseline; RCT3, complete outcome data; RCT4, outcome assessors blinded to intervention; RCT5, participants adhered to assigned intervention.

| **Ref.** | **First author, year** | **Study design** | **S1** | **S2** | **RCT1** | **RCT2** | **RCT3** | **RCT4** | **RCT5** | **Main methodological concerns** | **Overall interpretation** |
| --- | --- | --- | --- | --- | --- | --- | --- | --- | --- | --- | --- |
| 41 | Shin, 2025 | Rapid randomized quality-improvement trial | Y | Y | Y | CT | N | N | N | Small open-label trial; baseline imbalance could not be fully excluded; incomplete survey data; variable adherence and one intervention participant stopped using the tool. | Some concerns |
| 52 | Chowdhury, 2026 | Open-label randomized crossover trial | Y | Y | Y | Y | Y | N | CT | Open-label volunteer trial; no scribe-free washout; possible carryover/order effects; short periods and tool upgrades during the study. | Some concerns |
| 60 | Wendt, 2025 | Randomized step-wedge controlled study | Y | Y | Y | CT | CT | N | CT | Randomized step-wedge design with objective documentation metadata, but blinding was not possible; baseline comparability, missing data, and adherence details were incompletely clear. | Some concerns |
| 67 | Lukac, 2025 | Three-group pragmatic randomized clinical trial | Y | Y | Y | Y | CT | N | N | Strong randomized design with usual-care control and objective primary EHR outcome, but open-label, short-term, low actual use rates, and subjective secondary outcomes limit certainty. | Some concerns |
| 73 | Webb, 2026 | Single-site randomized crossover ED study | Y | Y | Y | Y | Y | N | CT | Randomized crossover design and high completion were strengths, but small single-site sample, short periods, no washout, order/carryover effects, and self-reported outcomes limit certainty. | Some concerns |

**Quantitative non-randomized studies (n = 17)**

QN1, participants are representative of the target population; QN2, measurements are appropriate regarding both outcome and intervention/exposure; QN3, complete outcome data; QN4, confounders accounted for in design or analysis; QN5, intervention/exposure administered as intended.

| **Ref.** | **First author, year** | **Study design** | **S1** | **S2** | **QN1** | **QN2** | **QN3** | **QN4** | **QN5** | **Main methodological concerns** | **Overall interpretation** |
| --- | --- | --- | --- | --- | --- | --- | --- | --- | --- | --- | --- |
| 39 | Misurac, 2025 | Pre-post observational pilot | Y | Y | N | Y | Y | N | Y | Single-center, short-term, small self-selected volunteer sample; no concurrent control; limited confounding control. | Substantial concerns |
| 40 | Owens, 2024 | Observational cohort survey plus retrospective pre-post subgroup analysis | Y | Y | Y | Y | CT | Y | Y | Non-random high-versus-low use comparison; documentation-time subgroup was small; residual/unmeasured confounding remains possible. | Some concerns |
| 45 | Wright, 2025 | Prospective observational trainee pilot | Y | Y | N | Y | N | N | Y | Non-random selected trainee sample; short follow-up; no control group; differential survey nonresponse and incomplete EHR metrics. | Substantial concerns |
| 46 | Stults, 2025 | Quality-improvement before-after evaluation | Y | Y | N | Y | CT | Y | Y | Single organization; purposively selected clinicians including clinical leaders/informatics champions; no control group; paired survey completion was limited. | Some concerns |
| 48 | Kuiper, 2026 | Three-month telehealth before-after pilot | Y | Y | CT | CT | CT | N | Y | Representativeness, measurement validation, response completeness, and missing data handling were insufficiently clear; no control group; self-reported outcomes predominated. | Substantial concerns |
| 51 | Olson, 2025 | Multicenter quality-improvement pre-post study | Y | Y | CT | Y | CT | Y | Y | Voluntary recruitment through digital-health leaders; no control group; short 30-day follow-up; non-anonymous self-reported outcomes and possible early-adopter bias. | Some concerns |
| 53 | Shah, 2025 | Prospective quality-improvement pilot | Y | Y | N | Y | Y | N | Y | Single-center self-selected pilot; no concurrent control; short follow-up; novelty, expectation, and learning effects could not be controlled. | Some concerns |
| 54 | You, 2025 | Two-system pre-post survey study | Y | Y | N | Y | N | N | CT | Low response rates; voluntary participation; no control group; self-reported use; short follow-up and possible response/selection bias. | Substantial concerns |
| 56 | Albrecht, 2025 | Pre- and post-implementation quality-improvement survey | Y | Y | CT | CT | CT | CT | Y | Pre- and post-surveys were not consistently paired and used different anonymity conditions; subjective outcomes predominated; no control group; validated instruments were limited. | Substantial concerns |
| 59 | Furrukh, 2025 | Small pilot before-after study | Y | Y | N | Y | Y | N | Y | Only six residents from one program; no control group; rotation, workload, learning, and time-trend effects could not be controlled. | Substantial concerns |
| 61 | Pelletier, 2025 | Six-month pilot with pre-post and interrupted time-series analysis | Y | Y | CT | Y | CT | Y | Y | Large EHR-log dataset and ITSA were strengths, but pilot selection, lack of concurrent control, survey subsamples, and generalizability remain concerns. | Some concerns |
| 62 | Galloway, 2024 | Before-after implementation survey | Y | Y | N | CT | N | N | Y | Small paired sample; survey wording/response options changed between baseline and follow-up; no control group; subjective outcomes predominated. | Substantial concerns |
| 64 | Lee, 2025 | Longitudinal before-after implementation study | Y | Y | N | Y | Y | N | Y | Only eight emergency physicians at one hospital; no control group; workload and attitudes were self-reported; short follow-up. | Substantial concerns |
| 66 | Omon, 2025 | Prospective before-after comparative study | Y | Y | N | Y | Y | N | Y | Only 12 rehabilitation professionals; fixed conventional-then-AI order; no randomization or control; phase and learning effects could not be excluded. | Substantial concerns |
| 68 | Duggan, 2025 | Prospective single-group pre-post quality-improvement study | Y | Y | CT | Y | Y | CT | Y | Objective EHR metrics and broad specialty coverage were strengths, but recruitment was selective; no concurrent control; short five-week exposure. | Some concerns |
| 69 | Harvey, 2025 | Surgical outpatient preliminary pilot | Y | Y | N | Y | CT | N | Y | Surgical subgroup included only three surgeons; no control group; short pilot; several outcomes did not reach statistical significance. | Substantial concerns |
| 70 | Haberle, 2024 | Peer-matched controlled cohort study | Y | Y | CT | Y | CT | Y | CT | Matched controls and operational outcomes were strengths, but participation was partly self-nominated, engagement data were not comparable with controls, and DAX use was variable. | Some concerns |

**Quantitative descriptive studies (n = 1)**

QD1, sampling strategy relevant to research question; QD2, sample representative of target population; QD3, measurements appropriate; QD4, risk of nonresponse bias low; QD5, statistical analysis appropriate.

| **Ref.** | **First author, year** | **Study design** | **S1** | **S2** | **QD1** | **QD2** | **QD3** | **QD4** | **QD5** | **Main methodological concerns** | **Overall interpretation** |
| --- | --- | --- | --- | --- | --- | --- | --- | --- | --- | --- | --- |
| 47 | Schneider, 2026 | Anonymous survey before and after onboarding | Y | Y | Y | N | Y | CT | Y | Single health system; selected users of generative AI scribes; anonymous non-paired design; nonresponse bias could not be fully assessed. | Some concerns |

**Qualitative research (n = 4)**

QR1, qualitative approach appropriate; QR2, data collection methods adequate; QR3, findings adequately derived from data; QR4, interpretation sufficiently substantiated by data; QR5, coherence among data sources, collection, analysis, and interpretation.

| **Ref.** | **First author, year** | **Study design** | **S1** | **S2** | **QR1** | **QR2** | **QR3** | **QR4** | **QR5** | **Main methodological concerns** | **Overall interpretation** |
| --- | --- | --- | --- | --- | --- | --- | --- | --- | --- | --- | --- |
| 50 | Bundy, 2024 | Qualitative semistructured interview study | Y | Y | Y | Y | Y | Y | Y | Small sample restricted to primary care physician users; transferability beyond this setting may be limited. | Few concerns |
| 58 | Van Tiem, 2026 | Qualitative semistructured interview study | Y | Y | Y | Y | Y | Y | Y | Single academic medical center; findings mainly transferable to similar implementation settings. | Few concerns |
| 63 | Shah, 2025 | Qualitative interview study using RE-AIM/PRISM | Y | Y | Y | Y | Y | Y | Y | Single organization and pilot-participant sample; transferability may be limited, but qualitative methods were coherent. | Few concerns |
| 65 | Stults, 2026 | Qualitative quality-improvement interview study | Y | Y | Y | Y | Y | Y | Y | Voluntary interview participation may have selected clinicians with stronger experiences, but sampling and thematic analysis were appropriate. | Few concerns |

**Mixed-methods studies (n = 8)**

MM1, adequate rationale for mixed-methods design; MM2, effective integration of qualitative and quantitative components; MM3, outputs of integration adequately interpreted; MM4, divergences/inconsistencies between components adequately addressed; MM5, each component meets quality criteria of the relevant tradition.

| **Ref.** | **First author, year** | **Study design** | **S1** | **S2** | **MM1** | **MM2** | **MM3** | **MM4** | **MM5** | **Main methodological concerns** | **Overall interpretation** |
| --- | --- | --- | --- | --- | --- | --- | --- | --- | --- | --- | --- |
| 42 | Guo, 2026 | Mixed-methods quality-improvement pilot | Y | Y | Y | Y | Y | CT | N | Uncontrolled self-selected implementation; two vendor platforms combined; qualitative analysis was light-touch and mainly based on open-ended survey comments. | Some concerns |
| 43 | Rabbani, 2025 | Mixed-methods pediatric primary care study | Y | Y | Y | Y | Y | Y | CT | Manually selected participants; custom survey instrument; no control group; qualitative component mainly based on free-text responses. | Some concerns |
| 44 | Alpert, 2025 | Mixed-methods pilot with interviews | Y | Y | Y | Y | Y | Y | N | Very small sample; no control group; clinicians selected from the highest 20% of after-hours EHR time, limiting generalizability. | Some concerns |
| 49 | Evans, 2025 | Mixed-methods allied health private-practice study | Y | Y | Y | Y | Y | CT | CT | Single private-practice organization; convenience sample; self-reported documentation time; no control group; qualitative transferability may be limited. | Some concerns |
| 55 | Nguyen, 2023 | Mixed-methods longitudinal pilot | Y | Y | Y | Y | Y | CT | N | Clinician-champion sample; extremely small survey/interview samples; no control group; one-month follow-up. | Some concerns |
| 57 | van Linschoten, 2026 | Prospective multicentre multi-perspective before-after mixed-methods study | Y | Y | Y | Y | Y | Y | CT | Strong use of direct observation and multiple perspectives, but only 12 GP/GP trainees were observed for short baseline/intervention periods; non-random before-after design. | Some concerns |
| 71 | Marquis, 2026 | Cross-sectional mixed-methods pilot survey | Y | Y | Y | Y | Y | Y | CT | Small single-system ED survey; cross-sectional self-report; qualitative component based on free-text responses with limited depth. | Some concerns |
| 72 | McCrudden, 2026 | Retrospective observational mixed-methods platform evaluation | Y | Y | Y | Y | Y | CT | CT | Large platform dataset, but no control group; internal observational data; low optional feedback rate; productivity changes may reflect external factors. | Some concerns |
