# Supplementary material for: Evidence on artificial intelligence-assisted clinical documentation and healthcare workers’ emotional wellbeing at work: a scoping review
Source: Front Psychol. 2026 Jun 24;17:1840884. doi: 10.3389/fpsyg.2026.1840884 (PMC13341515; doi:10.3389/fpsyg.2026.1840884)
Supplement: Supplementary file 6 [file Table_6.DOCX]

**Supplementary Table 6:** Reported funding sources, vendor involvement, and declared conflicts of interest across included studies.

| **Ref.** | **First author, year** | **Reported funding/support** | **Reported vendor involvement or implementation context** | **Declared COI/author relationship** | **Interpretation for evidence calibration** |
| --- | --- | --- | --- | --- | --- |
| 39 | Misurac, 2025 | Not reported/unclear | Commercial ambient AI documentation tool used in an institutional pilot | Not reported/unclear | Commercial implementation context present, but disclosures were unclear |
| 40 | Owens, 2024 | Not reported/unclear | Commercial DAX ambient voice technology used in health-system implementation. | Not reported/unclear | Commercial implementation context present |
| 41 | Shin, 2025 | Not reported/unclear | DAX Copilot selected partly because of prior institutional relationship with Nuance; vendor training videos/support were used | Not reported/unclear | Commercial implementation and vendor-support context relevant to interpretation |
| 42 | Guo, 2026 | No specific grant from public, commercial, or not-for-profit sectors | Institutional QI pilot using commercial ambient AI tools | No competing interests declared | Commercial tools used, but no specific funding or COI declared |
| 43 | Rabbani, 2025 | Supported by U.S. National Library of Medicine T15LM007092 | Commercial AI scribe used; vendor-supplied patient survey instrument reported | One author disclosed consulting fees from Abridge | Public support plus disclosed vendor-related consulting |
| 44 | Alpert, 2025 | Not reported/unclear | Commercial AI scribe implemented in primary care pilot | Not reported/unclear | Commercial implementation context present, but disclosures were unclear |
| 45 | Wright, 2025 | Not reported/unclear | Institutional trainee pilot using commercial Abridge ambient AI scribe | Not reported/unclear | Commercial implementation context present |
| 46 | Stults, 2025 | Funded by Sutter Health | Sutter disclosed as a strategic investor in Abridge; Abridge/Epic contributions acknowledged | One author reported personal fees from Intrinsic Brands outside the submitted work | Strong commercial and institutional implementation context requiring cautious interpretation |
| 47 | Schneider, 2026 | No funding declared | Commercial generative AI scribe implemented in health-system workflow | No competing interests declared | Commercial tool used, with no funding/COI declared |
| 48 | Kuiper, 2026 | HRSA support reported | Commercial AI scribe used in telehealth pilot | No competing financial interests declared. | Public funding plus commercial tool use |
| 49 | Evans, 2025 | No funding received | AI scribe implemented in a private allied health organization | Several authors were employed by the organization where the study took place | Organizational employment context relevant to interpretation |
| 50 | Bundy, 2024 | Not reported/unclear | Commercial DAX Copilot evaluated in a multi-site academic health system | Not reported/unclear | Commercial implementation context present |
| 51 | Olson, 2025 | Not reported/unclear | Abridge ambient AI scribe evaluated across multiple health systems | Abridge employment/leadership relationships disclosed for authors | Strong vendor-affiliation context requiring cautious interpretation |
| 52 | Chowdhury, 2026 | No external funding reported | Two commercial ambient AI scribe products compared in a randomized crossover trial | No competing interests declared | Commercial tools compared, with no external funding/COI declared |
| 53 | Shah, 2025 | No specific grant declared | Stanford institutional pilot using commercial DAX Copilot | Non-vendor COI disclosed for one author; no direct vendor COI clearly reported | Commercial tool use plus non-vendor COI disclosure |
| 54 | You, 2025 | Not reported/unclear | Ambient documentation technology used in institutional implementation studies | Not reported/unclear | Commercial/implementation context likely relevant, but disclosures were unclear |
| 55 | Nguyen, 2023 | Not reported/unclear | Commercial digital scribe/DAX tool implemented in a cancer center pilot | Not reported/unclear | Commercial implementation context present |
| 56 | Albrecht, 2025 | Not reported/unclear | Abridge platform evaluated in a QI survey | Abridge author affiliation disclosed | Vendor-affiliated authorship context relevant to interpretation |
| 57 | van Linschoten, 2026 | Funded by Rijnmond Dokters and Erasmus Trustfund | Rijnmond Dokters contributed to design, interpretation, and writing; external collaboration disclosed | First author reported funding and outside collaboration with Juvoly | Funder involvement and external collaboration should be considered. |
| 58 | Van Tiem, 2026 | No direct funding; institutional and P3 Strategic Initiatives support reported | Institutional ambient scribe pilot/rollout | No financial or nonfinancial competing interests declared | Institutional implementation context; no competing interests declared |
| 59 | Furrukh, 2025 | Not reported/unclear | DAX licenses used in a plastic surgery resident pilot | Not reported/unclear | Commercial implementation context present |
| 60 | Wendt, 2025 | No external funds/grants; DAX licenses provided by Nuance/Microsoft | Nuance/Microsoft provided limited software licenses; company reportedly not involved in study design, implementation, analysis, or writing | No known competing financial interests or personal relationships declared | Vendor-provided technology; stated vendor non-involvement |
| 61 | Pelletier, 2025 | Not reported/unclear | Business associate agreement with Abridge; digital scribe vendor and EHR usage data used | Not reported/unclear | Vendor/data infrastructure context relevant to interpretation |
| 62 | Galloway, 2024 | Not reported/unclear | Ambient listening documentation solution implemented in health system | Not reported/unclear | Commercial implementation context present, but disclosures were unclear |
| 63 | Shah, 2025 | Not reported/unclear | Qualitative study embedded in Stanford DAX Copilot pilot | Not reported/unclear | Commercial pilot context present |
| 64 | Lee, 2025 | Not reported/unclear | On-premise EHR-integrated LLM documentation assistant developed and implemented at Severance Hospital | Not reported/unclear | Internal tool context; commercial influence not clearly reported |
| 65 | Stults, 2026 | Not reported/unclear | Abridge platform used in Sutter pilot; qualitative interviews conducted after implementation | Not reported/unclear | Abridge implementation context present |
| 66 | Omon, 2025 | Not reported/unclear | Pleap Inc. contribution to generative AI documentation tool development acknowledged | Not reported/unclear | Developer contribution acknowledged; funding/COI unclear |
| 67 | Lukac, 2025 | Funded by UCLA Department of Medicine and others | DAX Copilot and Nabla compared in a pragmatic randomized trial | Not reported/unclear in extracted disclosure | Commercial comparative trial with institutional funding |
| 68 | Duggan, 2025 | Not reported/unclear | EHR-integrated commercial ambient scribe tool used in QI study | Not reported/unclear | Commercial implementation context present |
| 69 | Harvey, 2025 | Not reported/unclear | Commercial ambient AI scribe piloted in a medical group | Not reported/unclear | Commercial implementation context present |
| 70 | Haberle, 2024 | No funding declared | Nuance DAX implemented in a matched cohort study | No conflicts declared | Commercial tool use, with no funding/COI declared |
| 71 | Marquis, 2026 | Not reported/unclear | Commercial DAX Copilot used in an emergency department pilot | Not reported/unclear | Commercial implementation context present |
| 72 | McCrudden, 2026 | No external financial support or grants | Smart Notes developed and evaluated within Talkspace platform using operational data | All authors employed by Talkspace, LLC | Strong organizational/product-owner context requiring cautious interpretation |
| 73 | Webb, 2026 | Not reported/unclear | DAX and Abridge compared; vendor training/support provided for both tools | Not reported/unclear | Commercial comparative implementation context present |

Information was extracted as reported in the included articles. “Not reported/unclear” indicates that the relevant information was not clearly identifiable in the published article or available extracted text. Absence of a declared conflict of interest should not be interpreted as evidence that no commercial or implementation influence was present.
